# The implications of policy modeling assumptions for the projected impact of sugar-sweetened beverage taxation on body weight and type 2 diabetes in Germany

**DOI:** 10.1186/s12889-024-19488-5

**Published:** 2024-07-27

**Authors:** Karl M. F. Emmert-Fees, Andreea Felea, Matthias Staudigel, Jaithri Ananthapavan, Michael Laxy

**Affiliations:** 1 Professorship of Public Health and Prevention, TUM School of Medicine and Health, Technical University of Munich, Georg-Brauchle-Ring 60/62, 80992 München, Germany; 2 Institute for Medical Information Processing, Biometry, and Epidemiology, Pettenkofer School of Public Health LMU Munich, Munich, Germany; 3 Institute of Epidemiology, Research Center for Environmental Health, Helmholtz Zentrum München, Neuherberg, Germany; 4 TUM School of Management, Technical University of Munich, Munich, Germany; 5 Deakin Health Economics, Institute for Health Transformation, School of Health and Social Development, Deakin University, Geelong, Australia

**Keywords:** Sugar-sweetened beverages, Health taxation, Simulation modeling, Structural uncertainty, Health policy, Obesity, Type 2 diabetes

## Abstract

**Background:**

Evaluating sugar-sweetened beverage (SSB) taxation often relies on simulation models. We assess how assumptions about the response to SSB taxation affect the projected body weight change and subsequent health and economic impacts related to type 2 diabetes mellitus (T2DM) using Germany as an example.

**Methods:**

In the main analysis, we estimated changes in energy intake by age and sex under a 20% value-added tax on SSBs in Germany using marginal price elasticities (PE) and applied an energy equilibrium model to predict body weight changes. We then quantified the impact of several assumption *modifications*: SSB own-PE adjusted for consumption (M1)/based on alternative meta-analysis (M2); SSB consumption adjusted for underreporting (M3); substitution via marginal (M4a) or adjusted (M4b) cross-PE/as % of calorie change (M4c). We also assessed *scenarios* with alternative tax rates of 10% (S1) or 30% (S2) and including fruit juice (S3). We calculated overweight and obesity rates per *modification* and *scenario*. We simulated the impact on T2DM, associated healthcare costs, and disability-adjusted life years (DALYs) over the lifetime of the 2011 German adult population with a Markov model. Data included official demographics, national surveys, and meta-analyses.

**Results:**

A 20% value-added tax in Germany could reduce the number of men and women with obesity by 210,800 [138,800; 294,100] and 80,800 [45,100; 123,300], respectively. Over the population’s lifetime, this would lead to modest T2DM-related health and economic impacts (76,700 DALYs [42,500; 120,600] averted; €2.37 billion [1.33; 3.71] costs saved). Policy impacts varied highly across *modifications* (all in DALYs averted): (M1) 94,800 [51,500; 150,700]; (M2) 164,200 [99,500; 243,500]; (M3) 52,600 [22,500; 91,100]; (M4a) -18,100 [-111,500; 68,300]; (M4b) 25,800 [-31,400; 81,500]; (M4c) 46,700 [25,300; 77,200]. The variability in policy impact related to *modifications* was similar to the variability between alternative policy *scenarios* (all in DALYs averted): (S1) 26,400 [9,300; 47,600]; (S2) 126,200 [73,600; 194,500]; (S3) 342,200 [234,200; 430,400].

**Conclusions:**

Predicted body weight reductions under SSB taxation are sensitive to assumptions by researchers often needed due to data limitations. Because this variability propagates to estimates of health and economic impacts, the resulting structural uncertainty should be considered when using results in decision-making.

**Supplementary Information:**

The online version contains supplementary material available at 10.1186/s12889-024-19488-5.

## Background

Consistent evidence shows that the consumption of sugar-sweetened beverages (SSBs) contributes to poor diets and the global health and economic burden of non-communicable diseases (NCDs) [1]. SSB consumption is directly and indirectly associated with morbidity and mortality through overweight and obesity, dental caries, cancer, osteoarthritis, cardiovascular disease, and type 2 diabetes mellitus (T2DM) [2–6].

To reduce this burden, the taxation of SSBs has been proposed for many years [7, 8]. Depending on their objective, SSB taxes are designed to reduce SSB and sugar consumption, incentivize reformulation, and generate revenue to compensate for negative externalities through the associated disease burden [7, 9, 10]. Over 45 countries and jurisdictions have implemented taxes on SSBs of different magnitude and design (e.g., tiered vs. flat tax), and the World Health Organization (WHO) recommends taxation of SSBs as an essential preventive policy to achieve global NCD targets [11–13]. However, no such policy has been enacted in Germany [13].

Simulation models have been widely used to estimate the expected long-term health and economic impact of SSB taxation policies [14]. These models combine the best available epidemiological and economic evidence in a mathematical model to simulate policy scenarios compared to a counterfactual ‘do-nothing’ scenario. Results from modeling studies can, therefore, guide policymakers and promote effective NCD prevention [14–16].

However, the outputs of such models are subject to different sources (or types) of uncertainty. These arise, for example, from statistical estimation procedures, analytical decisions, simplifying assumptions, and relevant sub-group differences [17]. Conceptually, four sources of model output uncertainty are typically distinguished: stochastic uncertainty, parameter uncertainty, heterogeneity, and structural uncertainty [17]. Stochastic uncertainty (also called first-order uncertainty) is primarily relevant in simulation models where individuals are simulated separately (microsimulation) and describes the random variation in simulated individual outcomes. For example, for any number of individuals with the same probability of developing T2DM, not all will eventually get the disease in a simulation [17]. Parameter uncertainty (also called second-order uncertainty) describes the uncertainty related to the used model parameters. For example, if considered, the relative risk used to characterize the relationship between BMI and T2DM in a simulation is subject to uncertainty that propagates to the model outputs. Heterogeneity, on the other hand, describes any variation in model outputs that can be explained by population characteristics, such as age, sex, and others. For example, dietary intake, disease incidence, and/or relative risks linking the two may vary between men and women and across age groups [17].

Lastly, structural uncertainty is the uncertainty in outputs arising from multiple, potentially equally viable, model structures and related assumptions, of which typically only one is implemented for a particular analysis. For example, the effect of BMI on T2DM can be implemented with a continuous estimate (e.g., relative risk per unit of BMI) or by overweight and obesity status (e.g., separate relative risks for overweight and obesity compared to normal weight). Other aspects of structural uncertainty may include, for example, the selected modeling approach (e.g., Markov cohort model, microsimulation) and the included diseases and their relationships (e.g., in a model of the effects of BMI on coronary heart disease [CHD] and T2DM incidence, the latter could additionally be included as an independent risk factor for CHD) [14, 17].

In this study, we focus on the approach used to estimate the response to SSB taxation in a population which is one essential aspect of structural uncertainty in the context of the simulation-based evaluation of this type of policy. Concretely, the population response is the change in SSB intake and consequent change in sugar consumption following increased prices or reformulation due to an SSB tax. Because sugar is high in calories, a net reduction in sugar consumption, in theory, would lead to reduced overall caloric intake and, eventually, weight loss [18]. This long-term change in body weight is the most important model parameter for the simulated long-term health effects of SSB taxation. Therefore, considering the structural uncertainty in the link between the taxation policy and behavior change that results in SSB consumption change, including the compensatory consumption of other commodities (e.g., fruit juice), is critical for robust projections of long-term health benefits [14].

In this context, several aspects are important. First, most applied modeling studies typically implement the behavioral response to SSB taxes with price elasticities of demand, which quantify the (relative) change in SSB demand based on a (relative) change in price [14, 19]. However, a drawback of this approach is that researchers often assume the same marginal own- and cross-price elasticities for the whole population due to data limitations [14]. This disregard of behavioral heterogeneity may lead to an over- or underestimation of projected consumption changes. Economic studies based on high-dimensional consumer data have shown this is a critical assumption [20–22].

Additionally, researchers often face challenges in data availability and quality, which is particularly important in nutrition-related applications due to measurement biases such as underreporting [14, 23, 24]. The latter partly relates to parameter uncertainty and heterogeneity (e.g., the variation in mean SSB consumption per age-sex group) and structural uncertainty (e.g., the assumption that mean SSB consumption is underreported and the decision to account for this). In conjunction with the fact that price elasticities indicate relative changes in consumption, the resulting alternative assumptions have quantitative implications and can thus impact policy recommendations [25, 26].

Following the above considerations, this study aims to assess the structural uncertainty from a range of different assumptions regarding the population response to a hypothetical 20% value-added tax on SSBs in Germany with regard to the projected body weight reduction. To achieve this, we (1) test modifications of own- and cross-price elasticities of SSBs and fruit juice, which induce heterogeneity compared to a standard price elasticity approach, and (2) compare alternative approaches to implementing the effects of taxation in the model. We then use an established Markov cohort simulation model developed for the ACE-Obesity Policy study [27] and adapted to Germany to model the impact of the estimated weight reductions on T2DM and related healthcare costs. Lastly, we also explore different scenarios of tax rate and taxed beverage categories (SSBs-only vs. SSBs and fruit juice) to understand the relative importance of the structural uncertainty arising from the assessed policy modeling assumptions.

## Methods

### Study overview

**Fig. 1:**
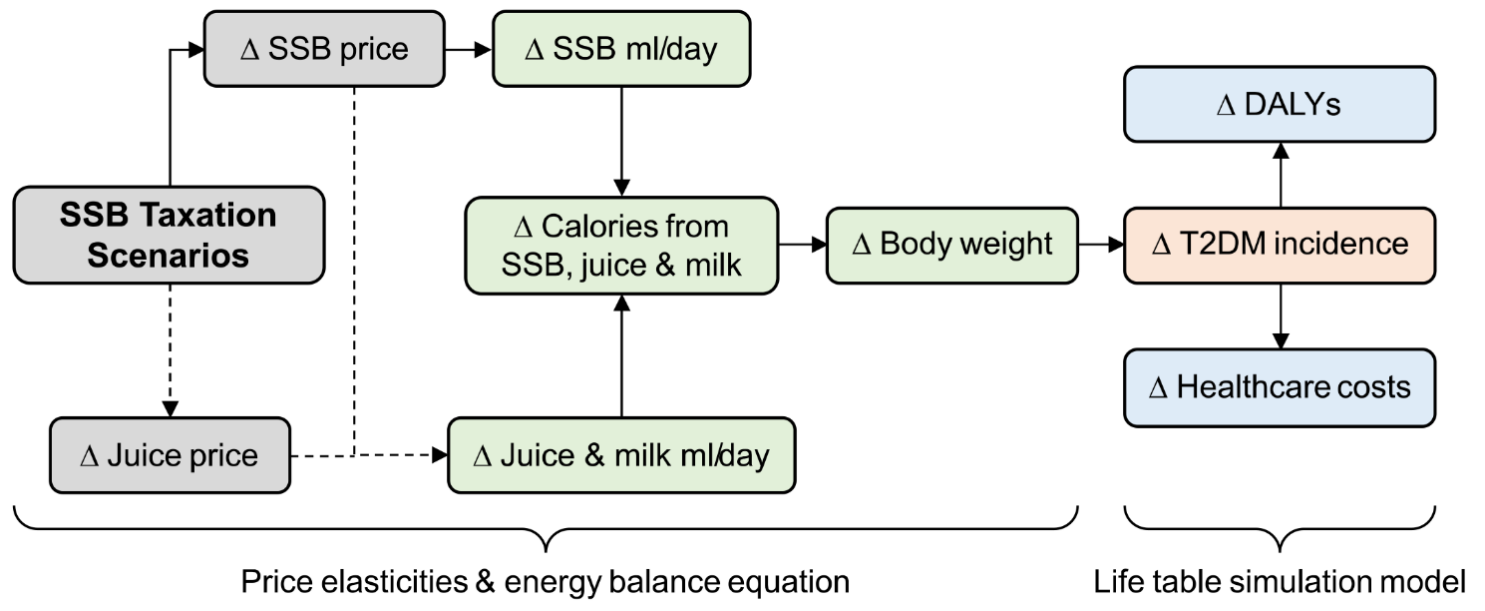
Logic model of the sugar-sweetened beverage tax simulation modeling approach. Abbreviations: DALY, disability-adjusted life years; ml, milliliter; SSB, sugar-sweetened beverages. Logic model of the simulation approach depicting the conceptual pathways of the analyses. Solid arrows indicate pathways of the main analysis. Dashed arrows indicate pathways that are relevant for scenario and modification analyses, such as substitution and additional taxation of fruit juice

Our general methodological approach to estimating the impact of SSB taxation in this study comprised several conceptual steps illustrated in Fig. 1. First, we derived the relative change in SSB prices. Second, we calculated the resulting change in SSB consumption using a standard price elasticity approach (see Sect. 2.3.2). Third, we estimated the long-term shift in the body weight (and consequently body mass index [BMI]) distribution resulting from changes in energy intake with established energy balance equations. Finally, we used a proportional multi-state life table Markov simulation model to analyze the long-term health and economic impacts on type 2 diabetes compared to a base case ‘do-nothing’ scenario.

We then repeated this analysis under alternative assumptions about how to correctly estimate the change in SSB consumption and energy intake under a tax, including the consideration of caloric substitution (hereafter: *modifications*; Sect. 2.3.3) to analyze the related structural uncertainty in the projected long-term BMI change and consequent T2DM-related outcomes. We additionally assessed alternative policy scenarios in which we varied the tax rate and considered the additional taxation of fruit juice (hereafter: *scenarios*; Sect. 2.3.4).

Simulation modeling analyses were conducted over the lifetime (i.e., maximum age of 100 years) of the 2011 German population aged 20 years and older, stratified by sex and 5-year age cohorts. We chose 2011 as the base year of our analysis and simulation model because most required data inputs were available for this or adjacent years, and we could not identify newer data sources. Although we acknowledge that our results are not representative of the current German population, this is not relevant to the quality of our analysis as we are primarily interested in the variability of estimated policy impacts due to structural uncertainty arising from policy modeling assumptions. Data sources included official German demographics, disease surveillance, and nationally representative dietary data. We provide an overview of input data and parameters in Appendix 1.

### Main taxation scenario

As the main scenario, we considered a hypothetical 20% value-added tax on SSBs in Germany, which is argued to be the minimum tax rate to substantially influence SSB purchasing and corresponds to the average rate of implemented taxes globally [28]. The tax was assumed to target SSBs, which are defined as all soft and fruit drinks with added caloric sweeteners, but not fruit juice without added sweeteners or artificially sweetened beverages, which is consistent with many implemented taxes [13]. While the WHO recommends that SSB tax rates should ideally take the sugar content of beverages into account to incentivize reformulation, and many implemented taxes, such as the Soft Drinks Industry Levy (SDIL) in the United Kingdom (UK) are designed accordingly, we chose not to analyze such a tax design due to data limitations [29].

Based on recent findings from a meta-analysis of outcomes under implemented SSB taxes, we assumed a tax pass-through from producers to consumers of 82% and that no relevant substitution to untaxed beverage categories would occur [11]. Thus, we do not account for the substitution to fruit juice or milk in the main analysis and explore the potential impact of this in detail via the implemented *modifications*. It is important to note that we are unable to consider the potential substitution to artificially sweetened beverages because the nutritional survey we use does not allow us to distinguish between ‘regular’ and low-calorie SSBs (see Sect. 2.3.1.) [30].

### Impact of SSB taxation on SSB consumption

Two key input parameters are needed to estimate the change in beverage consumption based on a taxation policy. First, the baseline level of consumption for all taxed beverage categories and potential substitutes is needed. Second, parameters indicating the demand for the relevant beverage categories based on price changes (i.e., price elasticities) – here induced by the tax – are needed to estimate changes in consumption based on the baseline level.

#### Baseline beverage consumption

We used baseline data on BMI (in kg/m²), consumption of SSBs, fruit juice and milk (all in milliliters [ml] per day), and total energy intake (in kilocalories [kcal] per day) from the second *German National Nutrition Survey* (NVS II), which is the most contemporary population-based data source of dietary intake in Germany [31], aggregated by sex and 5-year age cohorts using the appropriate survey weights (Appendix 1). Although the NVS II was conducted between 2005 and 2007, it is suitable for our purposes because we are not interested in population representativeness. Generally, Germany’s per capita consumption of different non-alcoholic beverage categories has remained stable [32]. Information on SSB sub-categories (e.g., low-calorie SSBs) and the number of calories consumed per beverage category are unfortunately not available in NVS II. We therefore assumed 48 kcal per 100 ml of SSB and 45 kcal per 100 ml of fruit juice based on a recent study from Canada, which estimated energy content based on sugar concentration in SSB sub-categories [33]. Considering the global scope of beverage production, this assumption is reasonable. For the average caloric value of milk, we used 59 kcal per 100 ml, which is based on a German scientific report on the energy content of milk, weighted for different levels of fat, and adjusted for density [34, 35]. Separate information on flavored milk products was not available as these were included in the overall milk category of the NVS II.

#### Standard price elasticity approach

We calculated consumption per beverage category after the tax using price elasticities (Appendix 2). Briefly, price elasticities measure the percentage change in demand for one good (e.g., SSBs) based on a 1% change in the price of the same good (own-price elasticity) or another good (cross-price elasticity).

We used estimates from an international meta-analysis of SSB own-price elasticities and cross-price elasticities of potential substitute beverages (Cabrera Escobar et al., 2013) primarily based on economic demand modeling studies [36]. We opted to use this study because it enabled us to use both own- and cross-price elasticities for SSBs and their potential substitutes from the same source [36]. This would not have been possible using estimates from a more recent meta-analysis of the SSB own-price elasticity (Andreyeva et al., 2022) based on ex-post evaluations using observational data in jurisdictions with implemented SSB taxes [11]. Estimates of the SSB own-price elasticity are very similar between both meta-analyses despite being based on studies using different methods [11, 36]. Indeed, the estimates from Cabrera Escobar et al. (2013) are slightly lower, making our approach conservative [11, 36].

Thus, for the own-price elasticity of SSBs, we used a mean value of -1.299, and for the cross-price elasticities for SSBs to fruit juice and milk, we used mean values of 0.388 and 0.129 [36]. As described above, we assumed no substitution between beverage categories in the main scenario, therefore only considering the (average) own-price elasticity of SSBs.

#### *Modifications* of policy modeling assumptions

We applied various literature-informed modifications to assess structural uncertainty from modeling assumptions regarding the behavioral response to the SSB tax. First, we investigated alternative assumptions that primarily affect the change in SSB consumption, including simplistic adjustments of own-price elasticities to assess the potential impact of heterogeneity by age as a proxy for consumption (*Modifications 1–3*). Second, we investigated several ways of implementing substitution to fruit juice and milk (*Modifications 4a-4c*).

##### *Modifications 1–3*: assumptions affecting the change in SSB consumption

First, evidence shows that individuals with a high baseline consumption will react less elastic to the tax, possibly due to mechanisms related to addiction (see, for example, Etilé & Sharma (2015)) [20, 21]. To account for this, we adjusted the own-price elasticity of SSBs for the level of baseline SSB consumption in the respective age-sex cohort, taking advantage of the strong correlation between SSB consumption and age in the data (*Modification 1*). This adjustment resulted in lower elasticities for younger age groups (i.e., high SSB consumers) (Appendix 6). SSB own-price elasticities were adjusted with the following equation:

1 $$\:\:{\delta\:}_{\text{o}\text{w}\text{n},\:age}=\gamma\:\times\:{\delta\:}_{\text{o}\text{w}\text{n}}\times\:\frac{\overline{{\theta\:}_{sex}}}{{\theta\:}_{sex,\:age}}$$

where $\:{\delta\:}_{\text{o}\text{w}\text{n},\:age}$ is the adjusted age-specific own-price elasticity for SSBs, $\:{\delta\:}_{\text{o}\text{w}\text{n}}$ is the population level marginal own-price elasticity from [36], $\:\overline{{\theta\:}_{sex}}$ is the sex-specific mean consumption of SSBs, $\:{\theta\:}_{sex,\:age}$ is the age-sex-specific mean consumption of SSBs and $\:\gamma\:$ is a scaling factor set to 0.5, which ensures that the mean of $\:{\delta\:}_{\text{o}\text{w}\text{n},\:age}$ for all males and females is equal to $\:{\delta\:}_{\text{o}\text{w}\text{n}}$.

Second, we used an SSB own-price elasticity estimate from another alternative meta-analysis based on interventional and prospective observational studies (*Modification 2)*. Afshin et al. (2017) [37] estimated an own-price elasticity of 0.674, which resulted in a decrease in SSB consumption of 11.05% (for all age-sex cohorts) in our taxation scenario (pass-through of 82%).

Third, we adjusted SSB consumption for potential misreporting because predicted relative changes in SSB consumption directly depend on baseline SSB intake (see Sect. 2.3.2.). We based this adjustment on the ratio between industry-reported, export-adjusted SSB consumption per capita and the self-reported consumption levels in the NVS II (*Modification 3*) [32]. We found that industry-reported SSB consumption was approximately 1.86 times higher than self-reported consumption in the NVS II [32]. We thus multiplied self-reported SSB consumption with 1.86 under the simplifying assumption that misreporting patterns and measurement biases were the same in all age-sex cohorts (Appendix 6).

##### Modifications 4a-c: assumptions about substitution between beverage categories

Fourth, we considered substitution to fruit juice and milk via estimates of cross-price elasticities, which were extracted from the literature together with the own-price elasticities of SSBs (*Modification 4a*) [36].

Fifth, we adjusted the above cross-price elasticity of fruit juice following a previous study to reflect that SSB high-consumers might have a higher cross-price elasticity of fruit juice with respect to the price of SSBs (i.e., are more likely to substitute SSBs with fruit juice) (*Modification 4b*) [38] (Appendix 6). To achieve this, the respective cross-price elasticity was adjusted with the following equation:

2 $$\:{\delta\:}_{\text{c}\text{r}\text{o}\text{s}\text{s},age}={\delta\:}_{\text{c}\text{r}\text{o}\text{s}\text{s}}\times\:\frac{{\theta\:}_{sex,\:age}}{\overline{{\theta\:}_{sex}}}$$

where $\:{\delta\:}_{\text{c}\text{r}\text{o}\text{s}\text{s},\:age}$ is the adjusted age-specific cross-price elasticity for SSBs and fruit juice, $\:{\delta\:}_{\text{c}\text{r}\text{o}\text{s}\text{s}}$ is the marginal cross-price elasticity, $\:\overline{{\theta\:}_{sex}}$ is the sex-specific mean consumption of SSBs and $\:{\theta\:}_{sex,\:age}$ is the age-sex-specific consumption of SSBs. The cross-price elasticity for milk was not adjusted because its value is comparably small and overall consumption levels in the NVS II data are low making it much less important for caloric substitution compared to fruit juice.

Lastly, we applied an alternative approach to include substitution effects based on a previous study that assumed SSBs would be equally replaced by water, low-calorie SSBs, and fruit juice [39]. Combined with earlier estimates of the effect of replacing SSBs with other beverages on energy intake, this study assumed that 61% of the calories reduced under SSB taxation would be compensated for (*Modification 4c*) [39, 40].

#### Policy scenario analyses

To understand the relative importance of the assumptions underlying the above modifications with respect to the estimated body weight change, we additionally conducted three policy scenario analyses. First, we varied the tax level to 10% (*Scenario 1*) and 30% (*Scenario 2*). Second, we assumed that the tax would additionally apply to fruit juice, which is also high in free sugars and may have detrimental health effects on T2DM [41]. Due to a lack of data, we assumed the same average own-price elasticity as for SSBs (*Scenario 3*).

### Long-term change in body weight after the tax

Using the baseline and calculated post-tax consumption levels for each modification and scenario, we computed the resulting change in energy intake in kcal by age group and sex. We then estimated the age-sex-specific long-term population-level changes in body weight with an energy balance equation, which postulates that at the population level, a 1% decrease in total energy intake will lead to an approximately 0.7% reduction in body weight at equilibrium [42]. Uncertainty in weight change was assessed using a Monte Carlo approach with 2,000 iterations implemented in R version 4.2.0 [43], which takes stochastic uncertainty in mean beverage intake, price-elasticities, and pass-through into account and is detailed in Appendix 3. Based on this predicted change in body weight, we calculated absolute and relative changes in overweight and obesity, assuming a log-normal distribution of BMI [44].

### Long-term health economic impact

We modeled the long-term health impact of changes in population-level BMI on T2DM, associated disability-adjusted life years (DALYs), and healthcare costs using a proportional multi-state life table Markov (MSLT) cohort model [27, 45, 46]. The model uses potential impact fractions (PIF) to estimate the proportion of T2DM incidence attributable to overweight and obesity [44]. Details on this widely adopted modeling method are given in Appendix 4 and elsewhere [47–49].

The MSLT model is implemented in Microsoft Excel. Uncertainty from model parameters (second-order uncertainty) was assessed using the Excel add-in software “Ersatz” and “EpiGearXL” with 2,000 Monte Carlo iterations by sampling from appropriate probability distributions of key parameters (Appendix 5) [17]. Uncertainty in outcomes is presented as 95%-uncertainty intervals [50, 51].

#### Impact on type 2 diabetes Mellitus

We obtained the most recent data on the incidence and prevalence of T2DM by age and sex from a 2011 analysis of the German statutory health insurance and retrieved all-cause and T2DM mortality rates from the German Health Data Reporting System (*Gesundheitsberichterstattung des Bundes*, www.gbe-bund.de) (Appendix 1) [52]. We estimated disease parameters for which no information was available based on prevalence, incidence, and mortality rates with DISMOD II [53].

To calculate the PIF of the shift in the BMI exposure distribution on T2DM incidence, we used published relative risks for T2DM per BMI unit increase stratified by age (Appendix 1) [44, 54].

#### Disability-adjusted life years and healthcare costs

We calculated DALYs with a recently published disability weight for T2DM [55] and prevalent life years with disability per person (i.e., pYLD rate) from the Global Burden of Disease Study (GBD) [56] (Appendix 1). Estimates of the 2011 German population by age and sex were retrieved from the Human Mortality Database [57] (Appendix 1).

To calculate potential healthcare cost savings, we multiplied the number of prevalent T2DM cases by the German healthcare costs per T2DM case. Estimates of one-year per-capita healthcare costs for patients with and without T2DM were based on a recent study using data from Germany’s largest statutory health insurance [58] (Appendix 1). Cost values were deflated to 2011 levels using the official German price index for the health sector. Projected savings are net of increased healthcare costs from other diseases due to longer life expectancy. Healthcare costs and DALYs were discounted at a rate of 3% [59].

## Results

### Main analysis

**Fig. 2:**
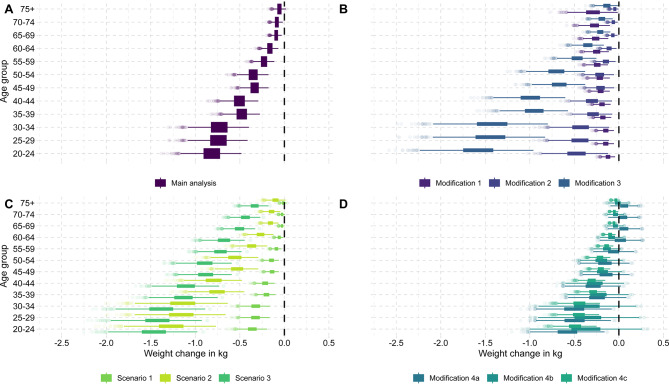
Body weight change based on different SSB tax scenarios and assumption modifications by age group for men. Abbreviations: kg, kilogram; SSB, sugar-sweetened beverages. Box plots display uncertainty in predicted body weight change based on 2,000 Monte Carlo simulations. The Monte Carlo sampling is based on the sample mean and standard error per beverage category from NVS II. The predicted change in energy intake using price elasticities and the corresponding long-term change in body weight based on Swinburn et al. (2009) is calculated per sample. Main analysis: Impact of 20% value-added tax on SSBs estimated with standard price elasticity approach (see Sect. 2.3.2.). Modification 1: SSB own-price elasticity adjusted for baseline SSB consumption (see Sect. 2.3.3.1.). Modification 2: SSB own-price elasticity from an alternative meta-analysis (see Sect. 2.3.3.1.). Modification 3: Baseline SSB consumption adjusted for underreporting (see Sect. 2.3.3.1.). Modification 4a: Including the substitution to fruit juice and milk with cross-price elasticities (see Sect. 2.3.3.2.). Modification 4b: Fruit juice cross-price elasticity adjusted for SSB consumption (see Sect. 2.3.3.2.). Modification 4c: Estimating substitution based on calories instead of cross-price elasticities (see Sect. 2.3.3.2.). Scenario 1: 10% value-added tax on SSBs (see Sect. 2.3.4.). Scenario 2: 30% value-added tax on SSBs (see Sect. 2.3.4.). Scenario 3: 20% value-added tax on SSBs and fruit juice (see Sect. 2.3.4.). The pass-through of the tax was set to 82% based on [11] in all analyses, modifications, and scenarios

**Fig. 3:**
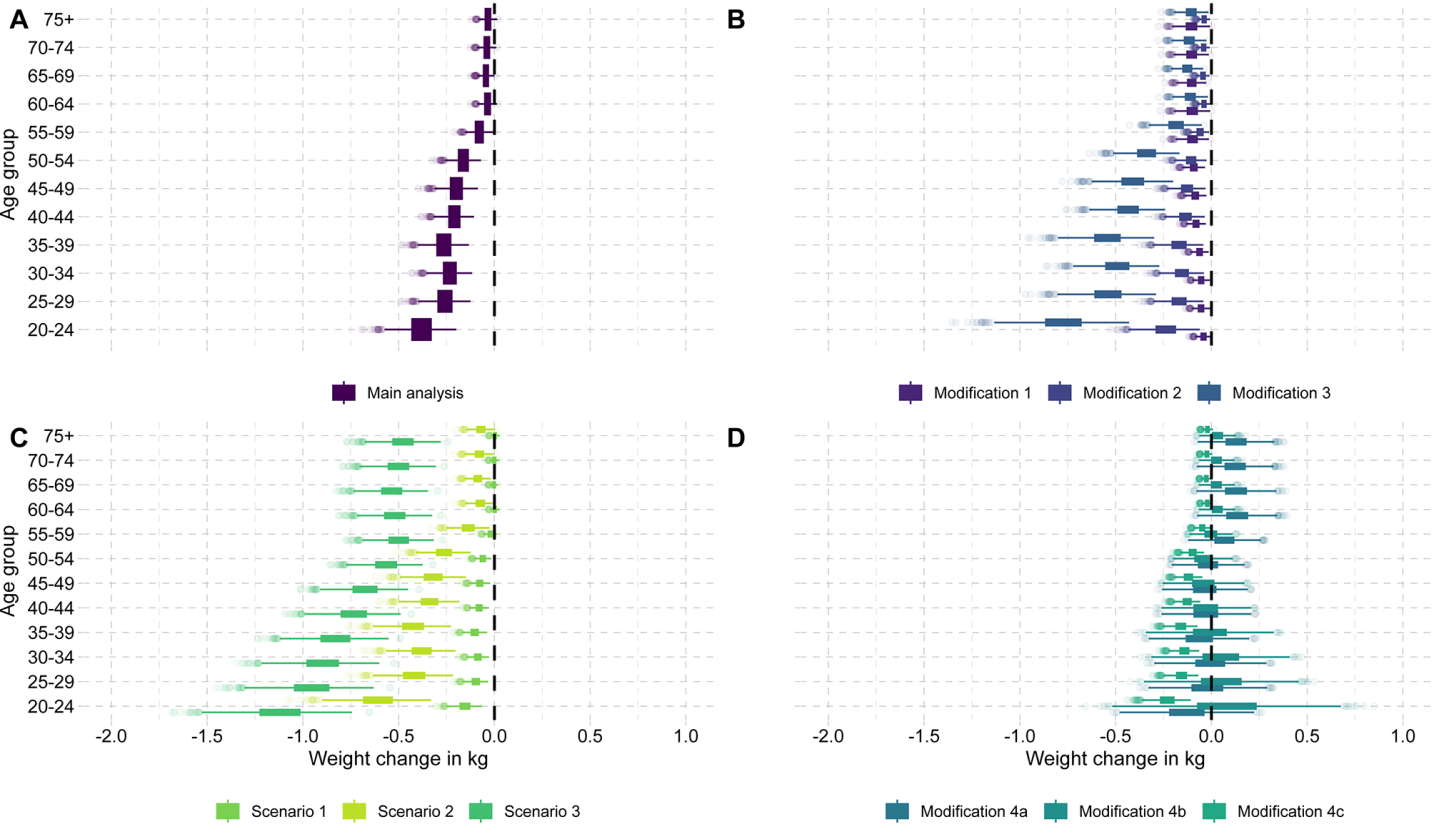
Body weight change based on different SSB tax scenarios and assumption modifications by age group for women. Abbreviations: kg, kilogram. Box plots display uncertainty in predicted body weight change based on 2,000 Monte Carlo simulations. The Monte Carlo sampling is based on the sample mean and standard error per beverage category from NVS II. The predicted change in energy intake using price elasticities and the corresponding long-term change in body weight based on Swinburn et al. (2009) is calculated per sample. Main analysis: Impact of 20% value-added tax on SSBs estimated with standard price elasticity approach (see Sect. 2.3.2.). Modification 1: SSB own-price elasticity adjusted for baseline SSB consumption (see Sect. 2.3.3.1.). Modification 2: SSB own-price elasticity from an alternative meta-analysis (see Sect. 2.3.3.1.). Modification 3: Baseline SSB consumption adjusted for underreporting (see Sect. 2.3.3.1.). Modification 4a: Including the substitution to fruit juice and milk with cross-price elasticities (see Sect. 2.3.3.2.). Modification 4b: Fruit juice cross-price elasticity adjusted for SSB consumption (see Sect. 2.3.3.2.). Modification 4c: Estimating substitution based on calories instead of cross-price elasticities (see Sect. 2.3.3.2.). Scenario 1: 10% value-added tax on SSBs (see Sect. 2.3.4.). Scenario 2: 30% value-added tax on SSBs (see Sect. 2.3.4.). Scenario 3: 20% value-added tax on SSBs and fruit juice (see Sect. 2.3.4.). The pass-through of the tax was set to 82% based on [11] in all analyses, modifications, and scenarios

In the main analysis, we observed moderate reductions in population body weight under a 20% value-added SSB tax in men and women compared to the base case without a tax (Figs. 2 and 3). Because SSB consumption is strongly associated with younger age and male sex and the response to the tax is proportional to consumption when using price elasticities, the largest long-term reduction in body weight of on average around 0.82 kg [95%-uncertainty interval: 0.57; 1.10] was predicted to occur in the cohort of men aged 20–24. In comparison, women aged 75 + are predicted to achieve only reductions of, on average, around 0.03 kg [0.00; 0.07] (Fig. 2).

Overall, the tax would lead to a reduction in the proportion of German men who are overweight and obese by 0.47 [0.32; 0.65] and 0.68 [0.45; 0.95] percentage points, respectively. This translates to 146,500 [99,200; 200,900] fewer men being overweight and 210,800 [138,800; 294,100] fewer being obese (Table 1). For women, the reduction would be 0.21 [0.13; 0.31] percentage points in overweight (69,300 [43,000; 100,300] fewer cases) and 0.25 [0.14; 0.38] percentage points in obesity (80,800 [45,100; 123,300] fewer cases) (Table 1).

**Table 1:** Reduction in overweight and obesity in Germany based on SSB taxation scenarios and assumption modifications

Main analysis	%-point reduction in proportion of overweight (95%-UI)	%-point reduction in proportion of obese (95%-UI)	Reduction in cases of overweight (95%-UI)	Reduction in cases of obese (95%-UI)
Men	0.47 (0.32; 0.65)	0.68 (0.45; 0.95)	146,500 (99,200; 200,900)	210,800 (138,800; 294,100)
Women	0.21 (0.13; 0.31)	0.25 (0.14; 0.38)	69,300 (43,000; 100,300)	80,800 (45,100; 123,300)
**Modifications**				
*Modification 1*				
Men	0.10 (0.06; 0.15)	0.49 (0.29; 0.73)	31,900 (18,600; 47,700)	151,600 (90,200; 225,300)
Women	0.07 (0.03; 0.12)	0.18 (0.08; 0.29)	22,300 (10,100; 37,200)	57,800 (26,300; 95,500)
*Modification 2*				
Men	0.28 (0.12; 0.45)	0.42 (0.19; 0.69)	85,600 (38,700; 140,000)	131,100 (59,600; 214,500)
Women	0.14 (0.06; 0.23)	0.18 (0.08; 0.30)	45,100 (20,100; 74,800)	57,500 (25,600; 97,100)
*Modification 3*				
Men	0.94 (0.65; 1.28)	1.34 (0.92; 1.82)	292,400 (200,500; 398,800)	416,800 (286,200; 566,800)
Women	0.45 (0.30; 0.63)	0.55 (0.35; 0.79)	145,200 (96,100; 203,200)	177,600 (112,400; 255,900)
*Modification 4a*				
Men	0.31 (0.12; 0.51)	0.29 (-0.12; 0.69)	94,900 (36,500; 158,800)	88,900 (-37,600; 213,900)
Women	0.02 (-0.16; 0.20)	-0.10 (-0.44; 0.21)	7,100 (-52,000; 64,900)	-32,500 (-142,000; 68,500)
*Modification 4b*				
Men	0.23 (-0.02; 0.48)	0.34 (-0.01; 0.71)	70,600 (-6,400; 150,600)	107,100 (-4,300; 219,600)
Women	-0.02 (-0.23; 0.19)	-0.02 (-0.28; 0.23)	-5,000 (-76,100; 63,000)	-6,500 (-92,000; 75,600)
*Modification 4c*				
Men	0.28 (0.18; 0.41)	0.42 (0.26; 0.61)	88,400 (56,900; 128,500)	129,300 (80,000; 190,300)
Women	0.13 (0.08; 0.20)	0.15 (0.08; 0.25)	42,200 (24,700; 64,100)	49,400 (26,300; 80,300)
**Scenarios**				
*Scenario 1*				
Men	0.20 (0.13; 0.29)	0.28 (0.17; 0.42)	63,100 (40,500; 89,300)	87,400 (51,200; 129,500)
Women	0.08 (0.04; 0.13)	0.08 (0.03; 0.15)	26,100 (13,400; 41,200)	26,200 (8,700; 47,200)
*Scenario 2*				
Men	0.75 (0.51; 1.02)	1.07 (0.72; 1.46)	232,200 (159,300; 317,100)	331,900 (225,200; 455,000)
Women	0.35 (0.22; 0.49)	0.42 (0.25; 0.61)	112,600 (72,700; 159,600)	134,900 (81,500; 198,600)
*Scenario 3*				
Men	0.97 (0.71; 1.26)	1.74 (1.29; 2.21)	302,000 (220,700; 391,500)	539,400 (401,700; 686,400)
Women	0.78 (0.58; 1.01)	1.21 (0.9; 1.55)	253,100 (187,200; 327,500)	392,100 (290,700; 503,300)

Abbreviations: SSB, sugar-sweetened beverages; UI, Uncertainty interval. Main analysis: Impact of 20% value-added tax on SSBs estimated with standard price elasticity approach (see Sect. 2.3.2.). Modification 1: SSB own-price elasticity adjusted for baseline SSB consumption (see Sect. 2.3.3.1.). Modification 2: SSB own-price elasticity from an alternative meta-analysis (see Sect. 2.3.3.1.). Modification 3: Baseline SSB consumption adjusted for underreporting (see Sect. 2.3.3.1.). Modification 4a: Including the substitution to fruit juice and milk with cross-price elasticities (see Sect. 2.3.3.2.). Modification 4b: Fruit juice cross-price elasticity adjusted for SSB consumption (see Sect. 2.3.3.2.). Modification 4c: Estimating substitution based on calories instead of cross-price elasticities (see Sect. 2.3.3.2.). Scenario 1: 10% value-added tax on SSBs (see Sect. 2.3.4.). Scenario 2: 30% value-added tax on SSBs (see Sect. 2.3.4.). Scenario 3: 20% value-added tax on SSBs and fruit juice (see Sect. 2.3.4.). The pass-through of the tax was set to 82% based on [11] in all analyses, modifications, and scenarios

Over the cohort’s lifetime, this reduction in body weight would translate into modest impacts on the epidemiology of T2DM in Germany. Overall, the simulation predicted around 86,400 [42,600; 141,100] fewer incident cases of T2DM and over 2.27 million [1.26; 3.56] fewer prevalent years lived with the disease. This would translate into over 76,700 averted DALYs [42,500; 120,600] and healthcare cost savings of around €2.37 billion [1.33; 3.71] for the German statutory health insurance (Appendix 7).

### Impact of policy modeling assumptions

#### Assumptions affecting the change in SSB consumption

The first set of modifications we analyzed was related to assumptions affecting the projected change in SSB consumption under the hypothetical SSB taxation scenario (*Modifications 1–3*; Appendix 6). These analyses revealed that alternative assumptions for own-price elasticities, as well as the assumed baseline level of SSB consumption, might significantly impact the predicted change in body weight in men (Fig. 2) and women (Fig. 3).

Adjusting own-price elasticities for the level of SSB consumption (*Modification 1*) drastically decreased projected reductions in overweight and obesity for men (31,900 [18,600; 47,700] and 151,600 [90,200; 225,300] fewer cases) and for women (22,300 [10,100; 37,200] and 57,800 [26,300; 95,500] fewer cases) (Table 1). Similarly, implementing the policy via a meta-analytic estimate of the effect of observed price increases on SSB consumption (*Modification 2*) led to smaller body weight reductions compared to the main analysis (Figs. 2 and 3). However, correcting self-reported SSB consumption for potential underreporting (*Modification 3*) resulted in substantially higher body weight reductions and impacts on overweight and obesity for men (292,400 [200,500; 398,800] and 416,800 [286,200; 566,800] fewer cases, respectively) and women (145,200 [96,100; 203,200] and 177,600 [112,400; 255,900] fewer cases for overweight and obesity respectively) (Table 1; Figs. 2 and 3).

**Fig. 4:**
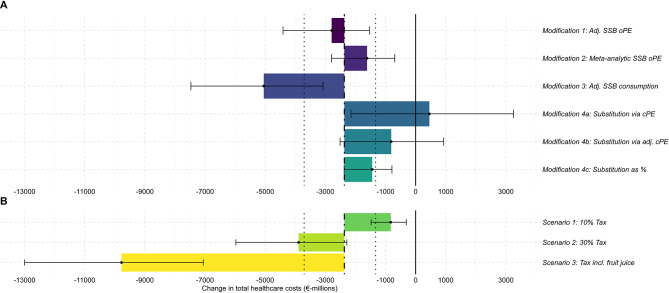
Healthcare costs saved under SSB tax in structural uncertainty analyses as ratio to results of main analysis. Panel A – Results from structural uncertainty analyses. Panel B – Results from policy scenario analyses. This plot shows the results of scenario and structural uncertainty analyses (colored bars = mean estimates; error bars = 95%-uncertainty intervals) in comparison to the mean healthcare costs saved in the main analysis (vertical dot-dashed line = mean estimate; vertical dotted lines = 95%-uncertainty interval). Main analysis: Impact of 20% value-added tax on SSBs estimated with standard price elasticity approach (see Sect. 2.3.2.). Modification 1: SSB own-price elasticity adjusted for baseline SSB consumption (see Sect. 2.3.3.1.). Modification 2: SSB own-price elasticity from an alternative meta-analysis (see Sect. 2.3.3.1.). Modification 3: Baseline SSB consumption adjusted for underreporting (see Sect. 2.3.3.1.). Modification 4a: Including the substitution to fruit juice and milk with cross-price elasticities (see Sect. 2.3.3.2.). Modification 4b: Fruit juice cross-price elasticity adjusted for SSB consumption (see Sect. 2.3.3.2.). Modification 4c: Estimating substitution based on calories instead of cross-price elasticities (see Sect. 2.3.3.2.). Scenario 1: 10% value-added tax on SSBs (see Sect. 2.3.4.). Scenario 2: 30% value-added tax on SSBs (see Sect. 2.3.4.). Scenario 3: 20% value-added tax on SSBs and fruit juice (see Sect. 2.3.4.). The pass-through of the tax was set to 82% based on [11] in all analyses, modifications, and scenarios

Consequently, Fig. 4 shows that these diverging predictions of body weight reduction implied considerable structural uncertainty in the projected health and economic impact regarding the prevention of T2DM. However, how these are propagated through the simulation model can be complex. For example, despite comparably little body weight reduction, T2DM prevention effects in *Modification 1* are larger than in the main analysis due to prevention at higher ages being more beneficial (Fig. 4, Appendix 7).

#### Assumptions about substitution between beverage categories

The second set of modifications we analyzed was related to assumptions affecting the potential caloric substitution to other beverages (*Modifications 4a-c*; Appendix 6). Here, the estimated impact of the analyzed SSB tax on body weight was considerably reduced (Figs. 2 and 3).

Using standard (i.e., unadjusted) cross-price elasticities (*Modification 4a*), the tax led to a slight reduction in overweight and obesity among men (94,900 [36,500; 158,800] and 88,900 [-37,600; 213,900] fewer cases) and even increases in obesity among women (32,500 [-68,500; 142,000] more cases) (Table 1). The latter results from “over-substitution” to juice in women above age 50 due to how price elasticities are applied in the standard approach. When adjusting cross-price elasticities (*Modification 4b*), this phenomenon was alleviated by reducing cross-price elasticities for low SSB consumers (change in obesity: 107,100 [-4,300; 219,600] fewer cases among men; 6,500 [-75,600; 92,000] more cases among women). Lastly, implementing substitution in terms of calories (*Modification 4c*) resulted in a slightly attenuated but still relevant decrease in body weight and prevented cases of overweight and obesity compared to the main analysis (men: 88,400 [56,900; 128,500] fewer cases of overweight and 129,300 [80,000; 190,300] fewer cases of obesity; women: 42,200 [24,700; 64,100] and 49,400 [26,300; 80,300] fewer cases) (Table 1; Figs. 2 and 3).

Again, this variability of predicted body weight reductions with respect to how substitution is considered leads to high structural uncertainty in the simulated lifetime health and economic impact related to the prevention of T2DM (Fig. 4, Appendix 7).

### Policy scenario analyses

When comparing alternative policy scenarios, we found that the projected change in body weight was expectedly sensitive to the tax rate (Figs. 2 and 3). In *Scenario 1*, reducing the tax rate to 10% led to a smaller reduction in cases of overweight and obesity for both men (63,100 [40,500; 89,300] and 87,400 [51,200; 129,500] fewer cases) and women (26,100 [13,400; 41,200] and 26,200 [8,700; 47,200] fewer cases) (Table 1). Conversely, increasing the tax rate to 30% in *Scenario 2* resulted in almost twice the amount of cases of overweight and obesity prevented in both men (232,200 [159,300; 317,100] and 331,900 [225,200; 455,000] fewer cases) and women (112,600 [72,700; 159,600] and 134,900 [81,500; 198,600] fewer cases) (Table 1). The additional taxation of fruit juice in *Scenario 3* resulted in the largest weight reduction among policy scenarios across all age-sex cohorts and the biggest reduction in overweight and obesity (Table 1; Figs. 2 and 3). The projected lifetime health and economic impacts in terms of DALYs and healthcare cost savings due to the corresponding prevention of T2DM were consistent with these findings (Fig. 4, Appendix 7).

## Discussion

### Summary

In this study, we assessed how structural uncertainty related to policy modeling assumptions might affect projected changes in body weight due to the introduction of a hypothetical 20% added-value tax on SSBs in Germany. Additionally, we used a cohort simulation model to estimate the resulting heterogeneity in the health and economic impact related to the subsequent prevention of T2DM.

In the main analysis, we projected that such a tax could lead to long-term reductions in population body weight, which were highest among the youngest age groups, particularly men, due to their high SSB consumption. Reductions in body weight ranged from 0.82 kg in men aged 20–24 to only 0.03 kg in women above 75. Overall, the modeled tax was associated with ~ 220,000 fewer cases of overweight and ~ 290,000 fewer cases of obesity. It would additionally prevent 2.27 million years lived with T2DM, avert 76,700 related DALYs, and save €2.37 billion in T2DM healthcare costs over the lifetime of the 2011 German population.

However, we showed that the predicted change in body weight and all subsequent outcomes, such as changes in obesity prevalence and impacts on T2DM, are highly variable regarding the modeling assumptions on how the SSB tax impacts behavior. We find that the variability in the prevented health burden under these assumptions is similar to the variability between alternative policy scenarios with different tax rates or taxed beverage categories. In particular, correctly specifying the baseline level of SSB consumption; whether assumed reductions in consumption are directly proportional to this baseline consumption level; and how potential mechanisms of caloric substitution are considered can have meaningful impacts on predicted changes in body weight and subsequently simulated health and economic outcomes.

### Comparison with other studies

In recent years, many studies have used simulation models to assess the health and economic impact of various diet policies, including the taxation of SSBs [14]. Some aspects of the complex effects of SSB taxation and their implications for policy evaluation, such as the relevance of substitution and different policy responses depending on pre-tax SSB consumption, have been analyzed by previous studies [20, 21, 60]. However, to our knowledge, this is the first study to comprehensively investigate how a range of common assumptions researchers make about these policies’ behavioral impact influences the findings from simulation studies.

In Germany, others have assessed the impact of SSB taxation on caries, overweight, and obesity alone or linked a hypothetical price increase of so-called “sin goods” (i.e., tobacco, red meat, and SSBs) by 50% to changes in the German Diabetes Risk Score to predict T2DM prevalence in 2040 [38, 61, 62]. These studies have also identified benefits of SSB taxation, although results are not directly comparable due to differences in modeling assumptions and disease pathways. Our results align with international modeling studies on SSB taxation, although direct quantitative comparisons are complicated by differences in policy scenarios, simulation techniques, populations, and time horizons [12, 48, 63, 64].

### Implications of policy modeling assumptions

We add to the literature on the simulation of SSB taxes by explicitly identifying, explaining, and assessing possible analytical decisions related to the implementation of the policy mechanism in simulation models (i.e., price increase leading to a change in energy intake) in the presence of uncertainty regarding the “true” impact of the policy on consumption. Economic studies using food purchasing data from consumer panels show that there may be considerable heterogeneity in the response to taxes on goods, which are detrimental to health [20, 22, 65–67]. Here, habit formation, addiction, health literacy, and psychological effects such as scarcity might affect changes in individual dietary behavior [68–70]. However, this complexity poses challenges to policy evaluation and is neither completely understood nor reflected in public health economic modeling studies of taxes on unhealthy foods and beverages [71]. Although we only perform simple adjustments to marginal price elasticities based on baseline SSB consumption levels (*Modification 1*), which are grounded in theory and findings from economic studies, we show that assumptions about this aspect of heterogeneity can have important implications for projected long-term health and economic outcomes and can be of a similar magnitude as design aspects of the simulated policy, such as the tax rate.

One issue of particular relevance for taxes that target specific foods (e.g., SSBs) is the possibility of consumers substituting with other untaxed foods within (e.g., fruit juice) or outside the respective category (e.g., sweets) [72, 73]. We show that the crude application of cross-price elasticities can lead to unrealistic results in some circumstances (*Modifications 4a-c*). We found that in cohorts with a low SSB and high fruit juice consumption (i.e., women in higher age groups), over-substitution from SSBs to fruit juice and, consequently, weight gain occurs due to relative changes in consumption [38]. To better predict the impact of taxation policies on population health, price elasticities disaggregated by sociodemographic characteristics, dietary habits, and weight status are required.

Although recent studies have concluded that there is no evidence for strong substitution to other beverage categories after introducing an SSB tax [11], some substitution of calories through various other food groups is likely to occur [73]. By comparing different assumptions regarding substitution in our simulation model, we show that failure to account for these effects can, in some cases, drastically overestimate, but not entirely eliminate, the potential projected public health benefits of SSB taxes. Additionally, we show that potential caloric substitutes of SSBs should be considered in the design of SSB taxes to maximize their impact. The correct specification of the response to price changes may also have important implications for other considerations relevant to the decision of policymakers for or against health taxes, such as the projected tax revenue.

However, we only analyze caloric substitution without accounting for the full complexity of diets and potentially relevant substitution patterns across food groups and between healthy and unhealthy foods, macronutrients, and micronutrients. Considering these aspects in future diet policy evaluations, including those of SSB taxes, could improve the validity of the respective simulations but would necessarily add complexity to the underlying epidemiological model and requires the availability of relevant input data [60].

We further show that the influence of limitations in dietary intake data from population surveys, typically used to simulate dietary policies, can be substantial. Because data collection in nutritional epidemiology is particularly prone to misreporting biases, modeling that relies on such data may also be biased [14]. Particularly in the case of sin goods, such as SSBs, underreporting can thus lead to an underestimation of the potential impact of policies.

### General implications for public health modeling

Although we only focus on one aspect of structural uncertainty in the simulation-based evaluation of SSB taxes, our study has broader implications for simulation modeling studies of the impact of public health policies. Compared to other areas in health modeling (e.g., the modeling of diabetes and its complications), potential structural uncertainties are often not considered or assessed in public health (economic) modeling studies [74]. However, our example illustrates that these uncertainties may have important impacts on the findings from modeling studies. Thus, health modelers should carefully analyze the respective decision context and highlight the structural mechanisms and assumptions in the simulation to which policy impacts are most sensitive. Because quantifying structural uncertainty with, for example, sensitivity analyses may not always be feasible, its potential implications should be at least reported and discussed. Our study further shows that it may sometimes be possible to approximate relevant complexity with additional simple assumptions, gauging its impact without the availability of respective quantitative data (*Modification 1*).

Beyond our considerations of structural uncertainty, we show that attempts to reduce complexity in the model-based evaluation of SSB taxes may lead researchers to make vastly different assumptions, which, ceteris paribus, can consequently lead to different findings. Thus, weighing whether to consider these or similar complexities in other public health policy evaluations is important and, if found to be relevant, will lead to better policy recommendations and decisions [75].

### Strengths and limitations

Our study has several strengths. This is the first study to rigorously assess how different assumptions on the behavioral effect of SSB taxation can influence predicted body weight reductions. We compare common policy modeling assumptions with simple modifications guided by theoretical considerations and seminal studies. Further, to assess the implications of the estimated long-term health and economic impact of SSB taxation on T2DM in Germany, we use an established simulation modeling framework, which has been used in various scenario modeling studies to evaluate diet policies.

However, this study has several limitations. First, while we consider heterogeneity in the policy response due to consumption levels, we are not able to account for other factors, such as, for example, income, which is associated with SSB consumption [1]. Second, we needed to rely on dietary intake and anthropometric data, which was collected between 2005 and 2007, and on T2DM epidemiological data for 2011. However, aggregate data suggests that the consumption of non-alcoholic beverage categories has stayed roughly constant over the last decade [32]. Additionally, our main conclusion regarding the structural uncertainty arising from policy modeling assumptions is not affected by the timeliness or representativeness of the data. Third, our nutritional survey does not contain product-level data, and we are unable to distinguish between ‘regular’ and low-calorie SSBs. This limits our ability to analyze different tax designs, such as various tax levels based on sugar content, related effects of reformulation as observed under the SDIL in the UK, and potential substitution effects to low-calorie SSBs [76]. We also do not account for all potential cross-price effects between SSBs and other beverages or foods, some of which might also be complements. However, while these aspects are important for analyzing the health and economic impacts of SSB taxation most accurately, they likely have only minor implications for our general conclusions regarding structural uncertainty related to policy modeling assumptions. Additionally, it remains unclear whether low-calorie SSBs indeed are an important substitute for SSBs [11, 21, 68, 77]. Fourth, our simulation approach only covers BMI as a risk factor and T2DM as an outcome, which ignores cancers and other cardiometabolic outcomes such as coronary heart disease. However, we explicitly do not aim to comprehensively simulate the health impact of SSB taxation scenarios in Germany; instead, we use this simple model to show the variability in simulation results that can be expected depending on assumptions about the policy mechanism. Fifth, the model assumes full effectiveness of the tax already in the first year of implementation and constant effectiveness in subsequent years, which likely leads to an overestimation of policy impacts. Sixth, we do not include quality of life related to BMI or cost categories beyond healthcare costs in our modeling, thus underestimating DALYs and economic effects from lost productivity, tax revenue, and administrative and legislative costs arising when introducing a taxation policy. Seventh, we do not incorporate uncertainty from several sources, such as T2DM epidemiology, all-cause mortality, and future trends in BMI. We also do not analyze other potentially important sources of structural uncertainty, such as the approach used to predict long-term changes in body weight. As described above, we purposefully restrict our analysis and use a simple model to discuss the implications of structural uncertainty related to assumptions about the policy mechanism. However, a systematic assessment of all possible policy modeling assumptions was outside the scope of this study. Finally, the impact of structural uncertainty on model outcomes is also likely dependent on the overall model structure and method. The simple Markov cohort model we used might be more sensitive than more complex models.

## Conclusions

Our study illustrates that predicted body weight reductions under SSB taxation are sensitive to assumptions made by researchers. Because this variability propagates to the simulated health and economic impact, for which BMI often is the key risk factor, the resulting structural uncertainty should be considered in simulation studies. As policies to reduce the obesity burden are urgently needed despite imperfect information, rigorous simulation studies can provide decision-makers with a range of possible outcomes under different policy scenarios. For future studies, data collection and the evidence underlying the behavioral response to health policies should be strengthened to reduce uncertainty concerning the long-term benefits of population-based preventive policies such as SSB taxes. Additionally, researchers should more transparently report and discuss relevant structural uncertainty in public health modeling studies. The results from this study can also serve as a reference for the structural uncertainty of SSB taxation impacts in evaluations that do not explicitly incorporate the implications of the assessed policy modeling assumptions.

### Electronic supplementary material

Below is the link to the electronic supplementary material.

Supplementary Material 1

## Data Availability

The data underlying this article was collected during the second German National Nutrition Study (*Nationale Verzehrstudie II*, NVS II) by the Max Rubner-Institute (MRI), Karlsruhe, Germany and was requested by the study authors for use in this article. The NVS II data can be requested for scientific purposes after application to the MRI under https://www.mri.bund.de/de/institute/ernaehrungsverhalten/forschungsprojekte/nvsii/scientific-use-file/. The R code for input data preparation and the Excel sheet for the main model will be shared on reasonable request to the corresponding author.

## Abbreviations

BMI: Body mass index
DALY: Disability–adjusted life year
MSLT: Proportional multi–state life table Markov model
NCDs: Non–communicable diseases
NVS II: Nationale Verzehrsstudie II (German National Nutrition Survey II)
pYLD: Prevalent life years with disability
SSB: Sugar–sweetened beverages
T2DM: Type 2 diabetes
WHO: World Health Organization
