# Supplementary material for: The implications of policy modeling assumptions for the projected impact of sugar-sweetened beverage taxation on body weight and type 2 diabetes in Germany
Source: BMC Public Health. 2024 Jul 27;24:2013. doi: 10.1186/s12889-024-19488-5 (PMC11283708; doi:10.1186/s12889-024-19488-5)
Supplement: Supplementary file 1 — Supplementary Material 1 [file 12889_2024_19488_MOESM1_ESM.docx]

**Supplementary information**

**The implications of policy modeling assumptions for the projected impact of sugar-sweetened beverage taxation on body weight and type 2 diabetes in Germany**

Karl M. F. Emmert-Fees*, Andreea Felea*, Matthias Staudigel, Jaithri Ananthapavan & Michael Laxy.

*These authors contributed equally.

**Appendix 1.** Input and baseline data tables

**Appendix 2.** Application of price elasticities

**Appendix 3.** Estimation of uncertainty in long-term change in body mass index

**Appendix 4.** Details of proportional multi-state life table simulation modeling

**Appendix 5.** Implementation of uncertainty analyses using Ersatz

**Appendix 6.** Details on modifications of policy modeling assumptions

**Appendix 7.** Health and economic impact of SSB tax scenarios and assumption modifications

**Appendix 8.** Overall health and economic impact for all analyses

**Appendix 1.** Input and baseline data tables

S1 Table: Overview of input data and parameters used in the simulation model

| **Data** | **Source** | **Assumptions & limitations** |
| --- | --- | --- |
| Baseline consumption of sugar-sweetened beverages, fruit juice and milk | NVS II [1] | Consumption patterns are assumed to be constant and can be extrapolated to 2011. |
| Baseline total energy intake, height, body weight and BMI | NVS II [1] | BMI is assumed to follow a log-normal distribution. Energy intake and anthropometrical variables are assumed to be constant and can be extrapolated to 2011. |
| Own- and cross-price elasticities of demand for sugar-sweetened beverages, fruit juice and milk | Cabrera Escobar et al. (2013) [2] | The published international values are assumed to be applicable to the 2011 German population. |
| Average calories per ml of fruit juice and sugar-sweetened beverage | Jones et al. (2018) [3] | Consumption patterns of beverage sub-categories and the resulting average caloric content of the overall category are assumed to be similar to Canada. |
| Caloric content of milk | Max Rubner-Institut (2014) [4] | Share of different types of milk are assumed to be applicable to NVS II consumption data. |
| Change in long-term body weight because of reduced energy intake | Swinburn (2009) [5] | The proposed energy balance model is assumed to accurately reflect long-term anthropometric changes in adult humans. |
| Relative risk of Type 2 Diabetes per unit of BMI | Mozaffarian et al. (2018) [6] | Etiologic relationship between BMI and Type 2 Diabetes is assumed to be transferable to the German population. |
| All-cause and diabetes-specific mortality rate | German Health Data Reporting System (*Gesundheitsberichterstattung des Bundes*, www.gbe-bund.de) | Deaths from diabetes as a contributing cause are not considered. |
| All-cause pYLD rate | IHME Global Burden of Disease Database [7] | Estimates published in the GBD database are based on modeling and subject to further assumptions. |
| Population count estimates by age and sex | The Human Mortality Database [8] | Values based on official statistics but corrected for intercensal projections. |
| Incidence and prevalence of Type 2 Diabetes | Schmidt et al. (2020) [9] | Non-SHI patients, which account for less than 20% of the German population, are not covered. |
| Case fatality of type 2 diabetes | Estimated with DisMod II [10] | Deaths from diabetes as a contributing cause are not considered. |
| Disability weight for uncomplicated Type 2 Diabetes | Technical documentation Global Burden of Disease 2019 [11] | Higher disability burden for complications of Type 2 Diabetes, such as retinopathy, amputations and end-stage renal disease is not accounted for. |
| Average healthcare costs for individuals with and without Type 2 Diabetes | Kähm et al. (2020) [12] | Non-SHI patients are not covered. Costs beyond the health sector are not included. |

Abbreviations: GBD, Global Burden of Disease; IHME, Institute for Health Metrics and Evaluation; NVS II, Nationale Verzehrsstudie II; pYLD, Prevalent years lived with disability; SHI, Statutory Health Insurance

S2 Table: Baseline distribution of body mass index by age and sex from NVS II [1]

|  | *Males* | | | *Females* | | |
| --- | --- | --- | --- | --- | --- | --- |
| *Age* | **Mean** | **SD** | **Average height** | **Mean** | **SD** | **Average height** |
| 20-24 | 23.89 | 3.91 | 1.80 | 23.20 | 4.25 | 1.65 |
| 25-29 | 25.43 | 4.28 | 1.80 | 24.07 | 4.80 | 1.67 |
| 30-34 | 25.63 | 4.17 | 1.79 | 24.51 | 5.00 | 1.66 |
| 35-39 | 26.40 | 3.83 | 1.79 | 24.99 | 5.28 | 1.66 |
| 40-44 | 26.97 | 4.17 | 1.79 | 25.86 | 5.27 | 1.66 |
| 45-49 | 27.36 | 4.10 | 1.78 | 26.25 | 5.20 | 1.65 |
| 50-54 | 27.69 | 4.50 | 1.77 | 26.87 | 5.64 | 1.64 |
| 55-59 | 28.29 | 4.15 | 1.75 | 27.02 | 5.11 | 1.63 |
| 60-64 | 28.63 | 4.38 | 1.75 | 27.53 | 5.30 | 1.62 |
| 65-69 | 28.42 | 3.92 | 1.74 | 28.33 | 5.03 | 1.60 |
| 70-74 | 28.10 | 3.81 | 1.72 | 28.18 | 4.79 | 1.59 |
| 75-79 | 28.92 | 3.86 | 1.71 | 28.46 | 4.75 | 1.57 |
| 80+ | 28.92 | 3.86 | 1.71 | 28.46 | 4.75 | 1.57 |

S3 Table: Baseline consumption of sugar-sweetened beverages (ml/day) by age and sex from NVS II [1]

|  | *Males* | | *Females* | |
| --- | --- | --- | --- | --- |
| *Age* | **Mean** | **SD** | **Mean** | **SD** |
| 20-24 | 475.68 | 717.54 | 197.76 | 402.89 |
| 25-29 | 394.48 | 681.91 | 135.72 | 304.12 |
| 30-34 | 394.79 | 780.98 | 128.03 | 301.29 |
| 35-39 | 245.43 | 481.19 | 133.21 | 386.23 |
| 40-44 | 248.62 | 501.15 | 99.02 | 293.38 |
| 45-49 | 168.33 | 416.81 | 93.45 | 365.37 |
| 50-54 | 168.61 | 452.36 | 76.16 | 268.13 |
| 55-59 | 107.31 | 280.10 | 42.48 | 234.48 |
| 60-64 | 80.05 | 258.35 | 26.79 | 155.45 |
| 65-69 | 49.13 | 182.73 | 29.29 | 141.95 |
| 70-74 | 43.67 | 164.53 | 27.37 | 122.30 |
| 75-79 | 30.79 | 163.43 | 25.28 | 108.82 |
| 80+ | 30.79 | 163.43 | 25.28 | 108.82 |

S4 Table: Baseline consumption of fruit juice (ml/day) by age and sex from NVS II [1]

|  | *Males* | | *Females* | |
| --- | --- | --- | --- | --- |
| *Age* | **Mean** | **SD** | **Mean** | **SD** |
| 20-24 | 360.38 | 573.81 | 339.53 | 526.64 |
| 25-29 | 342.71 | 538.01 | 311.12 | 487.00 |
| 30-34 | 330.75 | 542.72 | 305.78 | 452.38 |
| 35-39 | 307.62 | 453.70 | 247.21 | 402.27 |
| 40-44 | 274.17 | 450.84 | 212.47 | 373.12 |
| 45-49 | 248.85 | 413.08 | 194.03 | 323.16 |
| 50-54 | 236.30 | 415.43 | 161.06 | 310.44 |
| 55-59 | 200.71 | 336.88 | 165.88 | 293.05 |
| 60-64 | 208.54 | 384.11 | 183.63 | 326.85 |
| 65-69 | 165.49 | 320.85 | 181.90 | 308.56 |
| 70-74 | 137.97 | 252.67 | 172.76 | 294.30 |
| 75-79 | 104.17 | 204.57 | 164.49 | 284.41 |
| 80+ | 104.17 | 204.57 | 164.49 | 284.41 |

S5 Table: Baseline consumption of milk (ml/day) by age and sex from NVS II [1]

|  | *Males* | | *Females* | |
| --- | --- | --- | --- | --- |
| *Age* | **Mean** | **SD** | **Mean** | **SD** |
| 20-24 | 189.75 | 333.83 | 130.82 | 270.31 |
| 25-29 | 167.19 | 322.81 | 115.91 | 147.36 |
| 30-34 | 157.56 | 290.89 | 118.96 | 150.51 |
| 35-39 | 142.80 | 252.27 | 119.50 | 182.26 |
| 40-44 | 132.48 | 251.27 | 85.15 | 139.16 |
| 45-49 | 105.61 | 214.75 | 76.41 | 150.11 |
| 50-54 | 113.67 | 224.85 | 78.82 | 147.22 |
| 55-59 | 84.28 | 171.61 | 69.32 | 140.97 |
| 60-64 | 87.74 | 180.71 | 82.59 | 167.96 |
| 65-69 | 83.07 | 167.38 | 79.32 | 157.83 |
| 70-74 | 79.80 | 172.20 | 82.21 | 156.18 |
| 75-79 | 93.67 | 182.19 | 90.55 | 167.99 |
| 80+ | 93.67 | 182.19 | 90.55 | 167.99 |

S6 Table: Baseline energy intake (kcal/day) by age and sex from NVS II [1]

|  | *Males* | | *Females* | |
| --- | --- | --- | --- | --- |
| *Age* | **Mean** | **SD** | **Mean** | **SD** |
| 20-24 | 2954.79 | 1106.48 | 2005.34 | 658.89 |
| 25-29 | 2847.32 | 999.77 | 2045.50 | 672.86 |
| 30-34 | 2878.45 | 1222.47 | 2132.19 | 674.26 |
| 35-39 | 2743.60 | 911.53 | 2071.90 | 633.49 |
| 40-44 | 2703.49 | 861.58 | 1970.46 | 619.73 |
| 45-49 | 2710.03 | 934.30 | 1988.27 | 621.92 |
| 50-54 | 2572.66 | 860.88 | 1961.06 | 590.74 |
| 55-59 | 2436.25 | 780.62 | 1919.49 | 579.02 |
| 60-64 | 2425.50 | 783.98 | 1865.57 | 538.92 |
| 65-69 | 2350.19 | 671.43 | 1839.80 | 498.57 |
| 70-74 | 2211.20 | 610.30 | 1819.74 | 502.46 |
| 75-79 | 2158.00 | 581.07 | 1767.32 | 506.17 |
| 80+ | 2158.00 | 581.07 | 1767.32 | 506.17 |

S7 Table: Own-price elasticities for sugar-sweetened beverages (SSBs) and cross-price elasticities for juice and milk (Cabrera Escobar et al., 2013) [2]

| *Beverage category* | **Mean** | **Lower 95% CI** | **Upper 95% CI** |
| --- | --- | --- | --- |
| SSBs | -1.30 | -1.51 | -1.09 |
| Juice | 0.39 | 0.01 | 0.77 |
| Milk | 0.13 | -0.09 | 0.34 |

S8 Table: Relative risk per unit of body mass index for developing type 2 diabetes (Mozaffarian et al., 2018) [6]

| *Age* | **Mean RR** | **Lower 95% CI** | **Upper 95% CI** |
| --- | --- | --- | --- |
| 25-34 | 1.51 | 1.28 | 1.85 |
| 35-44 | 1.41 | 1.26 | 1.63 |
| 45-54 | 1.33 | 1.23 | 1.46 |
| 55-64 | 1.26 | 1.21 | 1.33 |
| 65-74 | 1.21 | 1.19 | 1.22 |
| 75+ | 1.10 | 1.08 | 1.13 |

S9 Table: All-cause mortality rates, prevalent years lived with disability (pYLD) rates and population size by age and sex of the 2011 German population

|  | *All-cause mortality rate* | | *Total pYLD rate* | | *Population size* | |
| --- | --- | --- | --- | --- | --- | --- |
| *Age* | **Males** | **Females** | **Males** | **Females** | **Males** | **Females** |
| 20 | 0.001 | 0.000 | 0.078 | 0.099 | 473,643 | 453,734 |
| 21 | 0.001 | 0.000 | 0.081 | 0.104 | 492,813 | 473,526 |
| 22 | 0.001 | 0.000 | 0.084 | 0.108 | 499,447 | 480,179 |
| 23 | 0.001 | 0.000 | 0.086 | 0.110 | 506,714 | 486,653 |
| 24 | 0.001 | 0.000 | 0.089 | 0.112 | 500,633 | 483,077 |
| 25 | 0.001 | 0.000 | 0.091 | 0.115 | 486,465 | 472,351 |
| 26 | 0.001 | 0.000 | 0.093 | 0.117 | 483,765 | 470,511 |
| 27 | 0.001 | 0.000 | 0.096 | 0.119 | 483,071 | 471,096 |
| 28 | 0.001 | 0.000 | 0.097 | 0.121 | 495,785 | 484,570 |
| 29 | 0.001 | 0.000 | 0.099 | 0.122 | 498,050 | 490,079 |
| 30 | 0.001 | 0.000 | 0.100 | 0.123 | 498,173 | 494,206 |
| 31 | 0.001 | 0.000 | 0.101 | 0.125 | 486,558 | 483,232 |
| 32 | 0.001 | 0.000 | 0.103 | 0.126 | 470,572 | 467,027 |
| 33 | 0.001 | 0.000 | 0.104 | 0.128 | 471,419 | 467,249 |
| 34 | 0.001 | 0.001 | 0.105 | 0.129 | 462,742 | 459,446 |
| 35 | 0.001 | 0.001 | 0.107 | 0.131 | 452,719 | 449,791 |
| 36 | 0.001 | 0.001 | 0.108 | 0.132 | 451,833 | 448,992 |
| 37 | 0.001 | 0.001 | 0.109 | 0.134 | 457,096 | 455,454 |
| 38 | 0.001 | 0.001 | 0.111 | 0.136 | 476,259 | 473,031 |
| 39 | 0.002 | 0.001 | 0.113 | 0.138 | 536,402 | 529,963 |
| 40 | 0.002 | 0.001 | 0.114 | 0.140 | 571,579 | 563,368 |
| 41 | 0.002 | 0.001 | 0.116 | 0.143 | 600,638 | 589,347 |
| 42 | 0.002 | 0.001 | 0.118 | 0.145 | 646,697 | 631,750 |
| 43 | 0.002 | 0.001 | 0.120 | 0.146 | 673,836 | 658,156 |
| 44 | 0.002 | 0.001 | 0.122 | 0.148 | 692,836 | 675,865 |
| 45 | 0.003 | 0.001 | 0.124 | 0.150 | 706,883 | 686,961 |
| 46 | 0.003 | 0.002 | 0.126 | 0.151 | 715,785 | 695,038 |
| 47 | 0.003 | 0.002 | 0.129 | 0.153 | 721,632 | 702,288 |
| 48 | 0.004 | 0.002 | 0.131 | 0.154 | 708,398 | 691,950 |
| 49 | 0.004 | 0.002 | 0.134 | 0.155 | 688,008 | 676,369 |
| 50 | 0.004 | 0.002 | 0.137 | 0.156 | 670,655 | 661,944 |
| 51 | 0.005 | 0.003 | 0.140 | 0.158 | 650,640 | 642,900 |
| 52 | 0.005 | 0.003 | 0.143 | 0.159 | 627,318 | 620,453 |
| 53 | 0.006 | 0.003 | 0.147 | 0.160 | 599,063 | 594,257 |
| 54 | 0.006 | 0.003 | 0.150 | 0.162 | 583,025 | 581,530 |
| 55 | 0.007 | 0.004 | 0.153 | 0.163 | 564,251 | 567,898 |
| 56 | 0.008 | 0.004 | 0.156 | 0.165 | 546,815 | 558,233 |
| 57 | 0.008 | 0.004 | 0.159 | 0.166 | 529,436 | 547,531 |
| 58 | 0.009 | 0.005 | 0.163 | 0.169 | 520,292 | 541,284 |
| 59 | 0.010 | 0.005 | 0.166 | 0.172 | 514,984 | 536,809 |
| 60 | 0.011 | 0.005 | 0.170 | 0.174 | 507,196 | 529,815 |
| 61 | 0.012 | 0.006 | 0.174 | 0.177 | 501,477 | 523,321 |
| 62 | 0.013 | 0.006 | 0.177 | 0.179 | 474,999 | 493,986 |
| 63 | 0.014 | 0.007 | 0.181 | 0.183 | 429,476 | 448,426 |
| 64 | 0.015 | 0.008 | 0.185 | 0.186 | 407,883 | 429,947 |
| 65 | 0.016 | 0.008 | 0.189 | 0.189 | 326,935 | 350,089 |
| 66 | 0.017 | 0.009 | 0.193 | 0.193 | 367,638 | 397,851 |
| 67 | 0.019 | 0.010 | 0.198 | 0.196 | 421,919 | 456,733 |
| 68 | 0.020 | 0.010 | 0.202 | 0.200 | 415,513 | 452,502 |
| 69 | 0.022 | 0.011 | 0.207 | 0.204 | 447,248 | 494,486 |
| 70 | 0.024 | 0.012 | 0.212 | 0.208 | 495,234 | 557,709 |
| 71 | 0.026 | 0.014 | 0.217 | 0.211 | 494,847 | 567,115 |
| 72 | 0.028 | 0.015 | 0.222 | 0.215 | 464,995 | 541,270 |
| 73 | 0.031 | 0.017 | 0.227 | 0.221 | 423,525 | 501,693 |
| 74 | 0.034 | 0.019 | 0.233 | 0.226 | 390,524 | 474,181 |
| 75 | 0.038 | 0.021 | 0.238 | 0.231 | 364,792 | 454,376 |
| 76 | 0.042 | 0.024 | 0.244 | 0.237 | 332,343 | 424,570 |
| 77 | 0.047 | 0.028 | 0.250 | 0.242 | 276,075 | 363,122 |
| 78 | 0.052 | 0.032 | 0.256 | 0.250 | 232,831 | 317,872 |
| 79 | 0.058 | 0.036 | 0.263 | 0.257 | 220,769 | 313,770 |
| 80 | 0.065 | 0.042 | 0.270 | 0.265 | 213,437 | 317,234 |
| 81 | 0.072 | 0.048 | 0.277 | 0.272 | 200,437 | 311,493 |
| 82 | 0.081 | 0.056 | 0.284 | 0.280 | 181,056 | 293,718 |
| 83 | 0.091 | 0.064 | 0.291 | 0.288 | 156,435 | 270,698 |
| 84 | 0.102 | 0.074 | 0.299 | 0.297 | 129,315 | 250,053 |
| 85 | 0.114 | 0.086 | 0.306 | 0.305 | 108,266 | 237,743 |
| 86 | 0.128 | 0.099 | 0.313 | 0.314 | 86,593 | 216,715 |
| 87 | 0.143 | 0.113 | 0.321 | 0.322 | 68,210 | 190,516 |
| 88 | 0.160 | 0.130 | 0.326 | 0.329 | 58,291 | 170,860 |
| 89 | 0.178 | 0.149 | 0.332 | 0.336 | 49,858 | 153,984 |
| 90 | 0.199 | 0.170 | 0.338 | 0.343 | 40,047 | 132,831 |
| 91 | 0.221 | 0.194 | 0.343 | 0.350 | 27,584 | 96,706 |
| 92 | 0.246 | 0.221 | 0.349 | 0.357 | 14,972 | 55,435 |
| 93 | 0.273 | 0.251 | 0.352 | 0.362 | 8,448 | 33,428 |
| 94 | 0.303 | 0.285 | 0.356 | 0.367 | 6,292 | 26,723 |
| 95 | 0.336 | 0.323 | 0.360 | 0.373 | 5,321 | 24,313 |

S10 Table: All-cause mortality rates, prevalent years lived with disability (pYLD) rates and population size by age and sex in the 2011 German population

|  | *Incidence rate* | | *Prevalence rate* | | *Mortality rate* | | *Case fatality rate* | |
| --- | --- | --- | --- | --- | --- | --- | --- | --- |
| *Age* | **Males** | **Females** | **Males** | **Females** | **Males** | **Females** | **Males** | **Females** |
| 20 | 0.000 | 0.000 | 0.000 | 0.000 | 0.000 | 0.000 | 0.000 | 0.000 |
| 21 | 0.000 | 0.000 | 0.000 | 0.000 | 0.000 | 0.000 | 0.000 | 0.000 |
| 22 | 0.000 | 0.000 | 0.000 | 0.000 | 0.000 | 0.000 | 0.000 | 0.000 |
| 23 | 0.000 | 0.000 | 0.000 | 0.000 | 0.000 | 0.000 | 0.000 | 0.000 |
| 24 | 0.000 | 0.000 | 0.000 | 0.000 | 0.000 | 0.000 | 0.000 | 0.000 |
| 25 | 0.000 | 0.000 | 0.000 | 0.000 | 0.000 | 0.000 | 0.000 | 0.000 |
| 26 | 0.000 | 0.000 | 0.000 | 0.000 | 0.000 | 0.000 | 0.000 | 0.000 |
| 27 | 0.000 | 0.000 | 0.000 | 0.000 | 0.000 | 0.000 | 0.000 | 0.000 |
| 28 | 0.000 | 0.000 | 0.000 | 0.000 | 0.000 | 0.000 | 0.000 | 0.000 |
| 29 | 0.000 | 0.000 | 0.000 | 0.000 | 0.000 | 0.000 | 0.000 | 0.000 |
| 30 | 0.000 | 0.000 | 0.000 | 0.000 | 0.000 | 0.000 | 0.000 | 0.000 |
| 31 | 0.000 | 0.000 | 0.000 | 0.000 | 0.000 | 0.000 | 0.000 | 0.000 |
| 32 | 0.000 | 0.000 | 0.000 | 0.000 | 0.000 | 0.000 | 0.000 | 0.000 |
| 33 | 0.000 | 0.000 | 0.000 | 0.000 | 0.000 | 0.000 | 0.000 | 0.000 |
| 34 | 0.000 | 0.000 | 0.001 | 0.000 | 0.000 | 0.000 | 0.000 | 0.000 |
| 35 | 0.000 | 0.000 | 0.001 | 0.001 | 0.000 | 0.000 | 0.000 | 0.000 |
| 36 | 0.000 | 0.000 | 0.001 | 0.001 | 0.000 | 0.000 | 0.000 | 0.000 |
| 37 | 0.001 | 0.000 | 0.002 | 0.001 | 0.000 | 0.000 | 0.000 | 0.000 |
| 38 | 0.001 | 0.000 | 0.002 | 0.001 | 0.000 | 0.000 | 0.000 | 0.000 |
| 39 | 0.001 | 0.001 | 0.003 | 0.002 | 0.000 | 0.000 | 0.000 | 0.000 |
| 40 | 0.001 | 0.001 | 0.005 | 0.002 | 0.000 | 0.000 | 0.000 | 0.000 |
| 41 | 0.002 | 0.001 | 0.006 | 0.003 | 0.000 | 0.000 | 0.000 | 0.000 |
| 42 | 0.002 | 0.001 | 0.008 | 0.004 | 0.000 | 0.000 | 0.000 | 0.000 |
| 43 | 0.003 | 0.002 | 0.010 | 0.006 | 0.000 | 0.000 | 0.000 | 0.000 |
| 44 | 0.003 | 0.002 | 0.013 | 0.007 | 0.000 | 0.000 | 0.000 | 0.000 |
| 45 | 0.004 | 0.002 | 0.016 | 0.009 | 0.000 | 0.000 | 0.000 | 0.000 |
| 46 | 0.004 | 0.002 | 0.019 | 0.011 | 0.000 | 0.000 | 0.000 | 0.000 |
| 47 | 0.005 | 0.003 | 0.023 | 0.014 | 0.000 | 0.000 | 0.002 | 0.000 |
| 48 | 0.005 | 0.003 | 0.027 | 0.016 | 0.000 | 0.000 | 0.002 | 0.000 |
| 49 | 0.006 | 0.003 | 0.031 | 0.019 | 0.000 | 0.000 | 0.002 | 0.000 |
| 50 | 0.006 | 0.004 | 0.035 | 0.022 | 0.000 | 0.000 | 0.002 | 0.000 |
| 51 | 0.007 | 0.004 | 0.039 | 0.025 | 0.000 | 0.000 | 0.002 | 0.000 |
| 52 | 0.007 | 0.005 | 0.044 | 0.029 | 0.000 | 0.000 | 0.002 | 0.000 |
| 53 | 0.008 | 0.005 | 0.049 | 0.032 | 0.000 | 0.000 | 0.002 | 0.000 |
| 54 | 0.008 | 0.005 | 0.055 | 0.036 | 0.000 | 0.000 | 0.002 | 0.000 |
| 55 | 0.009 | 0.006 | 0.061 | 0.040 | 0.000 | 0.000 | 0.002 | 0.000 |
| 56 | 0.010 | 0.006 | 0.067 | 0.044 | 0.000 | 0.000 | 0.002 | 0.001 |
| 57 | 0.010 | 0.007 | 0.073 | 0.049 | 0.000 | 0.000 | 0.002 | 0.001 |
| 58 | 0.011 | 0.008 | 0.080 | 0.054 | 0.000 | 0.000 | 0.002 | 0.001 |
| 59 | 0.012 | 0.008 | 0.088 | 0.059 | 0.000 | 0.000 | 0.002 | 0.001 |
| 60 | 0.013 | 0.009 | 0.096 | 0.065 | 0.000 | 0.000 | 0.002 | 0.001 |
| 61 | 0.014 | 0.010 | 0.105 | 0.071 | 0.000 | 0.000 | 0.002 | 0.001 |
| 62 | 0.015 | 0.010 | 0.114 | 0.078 | 0.000 | 0.000 | 0.002 | 0.001 |
| 63 | 0.015 | 0.011 | 0.123 | 0.085 | 0.000 | 0.000 | 0.002 | 0.001 |
| 64 | 0.016 | 0.012 | 0.133 | 0.092 | 0.000 | 0.000 | 0.002 | 0.001 |
| 65 | 0.017 | 0.012 | 0.142 | 0.100 | 0.000 | 0.000 | 0.002 | 0.002 |
| 66 | 0.018 | 0.013 | 0.152 | 0.108 | 0.000 | 0.000 | 0.002 | 0.002 |
| 67 | 0.018 | 0.014 | 0.161 | 0.116 | 0.000 | 0.000 | 0.003 | 0.002 |
| 68 | 0.019 | 0.014 | 0.170 | 0.124 | 0.001 | 0.000 | 0.003 | 0.002 |
| 69 | 0.020 | 0.015 | 0.178 | 0.132 | 0.001 | 0.000 | 0.003 | 0.002 |
| 70 | 0.021 | 0.016 | 0.186 | 0.140 | 0.001 | 0.000 | 0.003 | 0.002 |
| 71 | 0.021 | 0.016 | 0.194 | 0.148 | 0.001 | 0.000 | 0.003 | 0.002 |
| 72 | 0.022 | 0.017 | 0.202 | 0.155 | 0.001 | 0.000 | 0.003 | 0.002 |
| 73 | 0.022 | 0.017 | 0.210 | 0.163 | 0.001 | 0.000 | 0.004 | 0.003 |
| 74 | 0.023 | 0.018 | 0.218 | 0.171 | 0.001 | 0.001 | 0.004 | 0.003 |
| 75 | 0.023 | 0.019 | 0.225 | 0.179 | 0.001 | 0.001 | 0.004 | 0.003 |
| 76 | 0.024 | 0.019 | 0.232 | 0.187 | 0.001 | 0.001 | 0.005 | 0.004 |
| 77 | 0.025 | 0.020 | 0.238 | 0.195 | 0.001 | 0.001 | 0.005 | 0.004 |
| 78 | 0.025 | 0.020 | 0.245 | 0.203 | 0.001 | 0.001 | 0.005 | 0.005 |
| 79 | 0.026 | 0.021 | 0.250 | 0.210 | 0.002 | 0.001 | 0.006 | 0.005 |
| 80 | 0.026 | 0.021 | 0.255 | 0.218 | 0.002 | 0.001 | 0.007 | 0.006 |
| 81 | 0.026 | 0.021 | 0.260 | 0.225 | 0.002 | 0.002 | 0.007 | 0.007 |
| 82 | 0.027 | 0.022 | 0.264 | 0.232 | 0.002 | 0.002 | 0.008 | 0.008 |
| 83 | 0.027 | 0.022 | 0.267 | 0.238 | 0.002 | 0.002 | 0.009 | 0.009 |
| 84 | 0.028 | 0.023 | 0.270 | 0.244 | 0.003 | 0.002 | 0.010 | 0.010 |
| 85 | 0.028 | 0.023 | 0.273 | 0.250 | 0.003 | 0.003 | 0.011 | 0.011 |
| 86 | 0.028 | 0.023 | 0.274 | 0.255 | 0.003 | 0.003 | 0.012 | 0.013 |
| 87 | 0.028 | 0.023 | 0.276 | 0.259 | 0.004 | 0.004 | 0.013 | 0.014 |
| 88 | 0.028 | 0.023 | 0.276 | 0.262 | 0.004 | 0.004 | 0.015 | 0.016 |
| 89 | 0.028 | 0.023 | 0.276 | 0.265 | 0.004 | 0.005 | 0.016 | 0.018 |
| 90 | 0.028 | 0.023 | 0.275 | 0.268 | 0.005 | 0.005 | 0.018 | 0.020 |
| 91 | 0.027 | 0.022 | 0.273 | 0.269 | 0.005 | 0.006 | 0.019 | 0.022 |
| 92 | 0.027 | 0.022 | 0.271 | 0.270 | 0.006 | 0.006 | 0.021 | 0.023 |
| 93 | 0.027 | 0.022 | 0.269 | 0.271 | 0.006 | 0.007 | 0.022 | 0.025 |
| 94 | 0.027 | 0.022 | 0.266 | 0.271 | 0.006 | 0.007 | 0.023 | 0.027 |
| 95 | 0.027 | 0.022 | 0.263 | 0.271 | 0.006 | 0.007 | 0.023 | 0.027 |

S11 Table: Healthcare costs (Euro/person/year) in Germany for patients with and without diabetes (Kähm et al., 2020) [12]

| *Age* | **Type 2 Diabetes costs** | **Baseline healthcare expenditure** |
| --- | --- | --- |
| <50 | 2,388 | 1,019 |
| 50-59 | 2,409 | 1,483 |
| 60-69 | 2,440 | 2,140 |
| 70-79 | 2,528 | 3,156 |
| 80+ | 2,235 | 3,975 |

**Appendix 2.** Application of price elasticities

To implement the behavioral response to the sugar-sweetened beverage (SSB) taxation policy in the model, we used price elasticities of demand for different beverage categories from an international meta-analysis [2]. The consumption changes of SSBs, fruit juice and milk after the tax were calculated according to the following formula (here exemplary for SSB consumption):

$${SSB}_{post-tax}={SSB}_{pre-tax}\times(1+tax amount\times\frac{{PE}_{SSB}}{100})$$

Where PE is the own-price elasticity of demand in the case of changes in SSB consumption and the respective cross-price elasticity of demand in the case of fruit juice and milk. The tax amount is specified in percent (e.g., 20%).

**Appendix 3.** Estimation of uncertainty in long-term change in body mass index

To estimate the appropriate uncertainty in the predicted change in body mass index (BMI) after the tax based on the applied energy balance equation from Swinburn et al., 2009 [5], we used a Monte-Carlo sampling approach for each scenario, which was implemented in R. For this, we constructed seven independent streams of 2,000 randomly generated uniformly distributed quantiles (minimum = 0.001, maximum = 0.999).

First, to generate uncertainty in the mean consumption of each beverage category we used one stream per beverage category to sample from the quantile function of a zero-truncated normal distribution by age group and sex based on the strata-specific mean intake values and corresponding standard errors from NVS II. Correlation between beverage categories was not taken into account, which potentially leads to an overestimation of uncertainty that we accept.

Second, to include uncertainty in price elasticities we used one stream per price elasticity (own-price elasticity of SSBs, cross-price elasticity for SSBs and fruit juice, cross-price elasticity for SSBs and milk from Cabrera-Escobar et al., 2013 [2]) to sample from the quantile function of a normal distribution based on the mean elasticity values and standard errors calculated with the confidence-intervals supplied in the meta-analysis [2]. To implement uncertainty around adjusted price elasticities, we assumed the same relative variation around the group-specific mean estimate as for the unadjusted elasticities.

Third, to include uncertainty around the pass-through value from the meta-analysis by Andreyeva et al., 2022 [13], we used the last remaining stream to sample from the quantile function of a normal distribution again with the respective mean pass-through and the corresponding standard error calculated with the confidence-interval from the meta-analysis [13].

This procedure resulted in a dataset of 2,000 possible values for the calorie reduction (i.e., average change in energy intake due to the tax) per age-sex group per scenario. The empirical distribution of these values thus takes combined stochastic uncertainty from beverage intake, price-elasticities and pass-through into account. For each iteration, we then calculated the predicted mean change in BMI with the following energy balance equation from Swinburn et al., 2009 [5]:

$$Weight after tax=\left( \frac{energy intake after tax}{energy intake before tax} \right)^{0.712}\times weight before tax$$

The resulting empirical uncertainty distributions of weight change per age-sex group per scenario were then directly implemented in the simulation model in Microsoft Excel.

**Appendix 4.** Details of proportional multi-state life table simulation modeling

Each population age-sex cohort in the model progresses through a disease table comprised of three states: Healthy, diseased with type 2 diabetes, and dead from type 2 diabetes (Figure 1). The cycle duration is one year, and the transition probabilities vary with age. For each cycle, the transition probability from the healthy to the diseased state is given by the type 2 diabetes incidence rate for the respective age. The transition probability from the diseased to the dead state is the case fatality rate in the given year. The probability of transcending from the diseased to the healthy state is considered to be zero, as type 2 diabetes remission hardly occurs in the absence of bariatric surgery, which is assumed to be the case [14].

S1 Figure: Schematic illustrating the states considered in the present Markov model and the associated transition probabilities for one cycle (year)


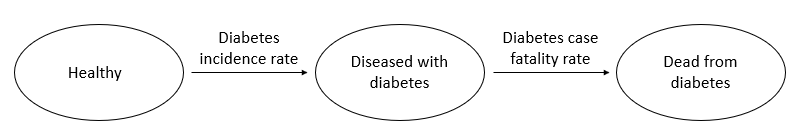


In epidemiological terms, a change in risk factor exposure results in a proportional change in the incidence of a related disease. The latter is captured by an effect measure known as potential impact fraction (PIF), which is closely related to the concept of population-attributable fractions. The relationship between the PIF value and incidence rate is shown in equation (1), where *I* stands for the initial incidence rate, and *I'* represents the new incidence rate after the change in risk factor exposure occurred [15].

|  | $I^{'}=I\left( 1-PIF \right)$ | (1) |
| --- | --- | --- |

There are several ways to compute PIF values. The “RR-method” is one reliable practice that is based on the change in a continuous relative risk (RR) associated with a specific categorical risk factor for a disease [15]. Equation (2) describes the PIF calculation according to the “RR-method”; *p_c_* stands for the population proportion associated with a given risk factor category *c*, *RR_c_* and *RR'_c_* represent the relative risks associated with the risk factor category *c* before and after the change in risk factor exposure occurred, respectively [15]. Notably, in equation (2), the relative risks change and not the population proportions, which remain the same before and after the change in risk factor exposure. This practice avoids over- or underestimating the PIF values that would otherwise be caused by non-linear artifacts.

|  | $PIF=\frac{\sum_{c=1}^{n} p_{c}{RR}_{c}-\sum_{c=1}^{n} p_{c}{RR^{'}}_{c}}{\sum_{c=1}^{n} p_{c}{RR}_{c}}$ | (2) |
| --- | --- | --- |

The relative risks in equation (2) can be computed based on a “per-unit” RR function. The form of the latter is expressed in equation (3), where *x* represents the mean exposure level of the risk factor in the respective category *c*, *a* is the per unit RR and *b* is the risk factor level for which *RR(x)* equals 1.

|  | $RR\left( x \right)=a^{x-b}$ | (3) |
| --- | --- | --- |

In the model, BMI is the categorical risk factor whose change leads to an adjustment of type 2 diabetes incidence. The risk factor categories are the BMI groups: normal weight, overweight and obese, such that *n* in equation (2) equals 3. The change in risk factor exposure is considered to occur after the SSBs tax is introduced, such that *RR_c_* and *RR'_c_* in equation (2) correspond to the baseline and the hypothetical population, respectively. A separate PIF value is calculated for each age-sex cohort based on their specific reduction in BMI after the SSB tax. The RR values in equation (2) are computed as functions of the mean BMI levels in the respective BMI categories as shown in equation (3). The constant *b* in equation (3) is the mean BMI level corresponding to the normal weight BMI (reference) category. The underlying assumption for the latter is that, for normal weight individuals, a one-point change in BMI leads to no change in type 2 diabetes risk. The base *a* in equation (3) represents the applied relative risk for developing type 2 diabetes associated with a one-point change in BMI [15].

As previously outlined, an important step in obtaining the PIF values was the calculation of population proportions corresponding to each BMI category. The scheme in Figure 2 illustrates the process of obtaining these population proportions and ultimately the individual PIF values for each cohort. For each age-sex cohort in the population, the values for the mean and standard deviation corresponding to a log-normal BMI distribution were first obtained (Figure 2).

S2 Figure: Schematic illustrating the stepwise approach to obtain the PIF value for each age-sex 5-year cohort in the hypothetical population


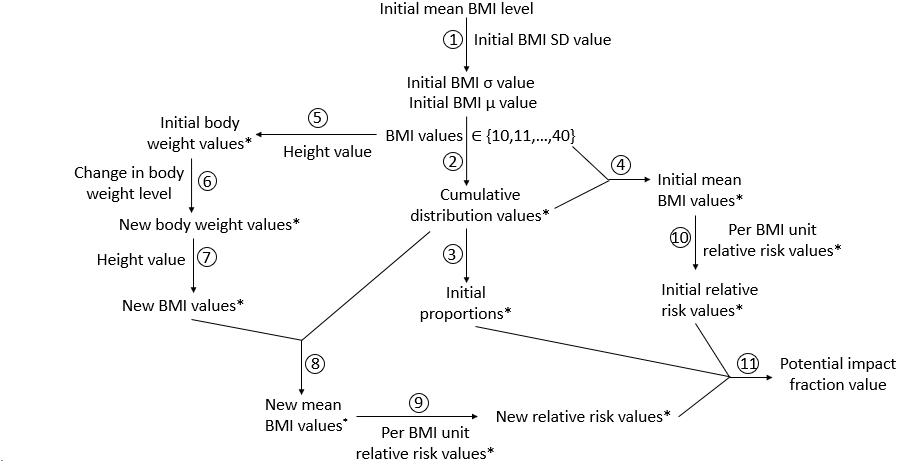


We assumed a log-normal distribution for BMI, because body size measures likely follow skewed distributions [16]. Given the mean BMI level and the standard deviation (SD) value corresponding to a normal BMI distribution, the values for the standard deviation σ and the mean µ corresponding to a log-normal BMI distribution can be computed as in equations (5) and (4), respectively.

|  | $\mu=\ln\left( mean \right)-0.5*\sigma^{2}$ | (4) |
| --- | --- | --- |
|  | $\sigma=\sqrt{ln({{SD}^{2}}/{{mean}^{2}+1)}}$ | (5) |

In the next stage, the previously obtained parameters µ and σ were used to derive the cumulative distribution values corresponding to individual integer BMI levels ranging from 10 to 40 (Figure 2, step 2). Knowing the cumulative distribution values corresponding to the BMI intervals [10, 25], (25, 30] and (30, 40], the population proportions corresponding to the normal weight, overweight and obese BMI categories could be computed for the baseline population (Figure 2). Once the population proportions were obtained, the mean BMI values corresponding to the respective BMI categories could be calculated, based on the general formula in equation (6). In the latter, *mean_BMIc_* is the average BMI value corresponding to the BMI category *c*, *i* represents the index corresponding to the BMI values ranging from 10 to 40, α is the cumulative distribution value corresponding to a given BMI level, and *p* is the population proportion in the respective BMI category.

|  | ${mean}_{{BMI}_{c}}=\frac{\sum_{i=1}^{n} \left( \alpha_{i+1}-\alpha_{i} \right)*0.5*({BMI}_{i}+{BMI}_{i+1})}{p_{c}}$ | (6) |
| --- | --- | --- |

The average BMI values for the different BMI categories of the hypothetical population were analogously computed. First, based on the mean height value of a given age-sex cohort, the baseline body weight levels corresponding to the BMI range 10 to 40 were calculated (Figure 2). The body weight change caused by the SSBs tax and corresponding to the respective cohort was then individually added to the baseline body weight values to derive the new body weight levels after tax implementation (Figure 2). The latter were further converted to BMI values by using the average height value of the respective cohort (Figure 2). Finally, these new BMI levels, together with the initial cumulative distribution values and the baseline population proportions were introduced in equation (6) to derive the new mean BMI levels in the respective BMI categories after tax (Figure 2) [15].

In a last stage, the mean BMI values obtained in equation (6) were used to derive the relative risks corresponding to the different BMI categories for the baseline and intervention populations, respectively (Figure 2). These relative risk values together with the previously derived proportions for the baseline population were used to obtain the PIF value for each cohort as in equation (2) (Figure 2). Notably, for each cohort, the per unit relative risk value used in equation (2) accounted for a random term drawn from a normal distribution as shown in equation (7). This was to introduce variability in the relative risk values attributed to the BMI categories of individual cohorts in uncertainty analyses. In equation (7), *a_λ_* is the per unit relative risk *a* adjusted for the random term λ, while the SE is the standard error.

|  | ${\ln(a}_{\lambda})=exp(\ln\left( a \right)+\lambda*{SE}_{ln(a)})$ | (7) |
| --- | --- | --- |

The natural logarithmic scale used in equation (7) accounts for assuming a log-normal distribution for the relative risk parameter, which agrees with good modelling practices [17].

Each age-sex cohort in the model has its own disease and a life table, respectively (Figure 3). The disease tables take as inputs yearly T2DM incidence and case fatality rates as well as T2DM disability weights. They return yearly T2DM prevalence and mortality rates as well as yearly rates for prevalent life years lived with disability (pYLD) (Figure 3). The newly obtained T2DM mortality and pYLD rates are then further used together with baseline all-cause mortality and pYLD rates to derive new all-cause mortality and pYLD rates (Figure 3). Using information on population size, the number of disability adjusted life years (DALYs) as well as the number of years lived can be computed for each cohort in both the baseline and intervention populations (Figure 3). The previously obtained values for the number of years of life lived are used to compute yearly numbers of T2DM incident cases, prevalent years, and deaths for each cohort in both populations (Figure 3).

S3 Figure: A schematic describing the second part of the modelling procedure for each age-sex cohort in the intervention population


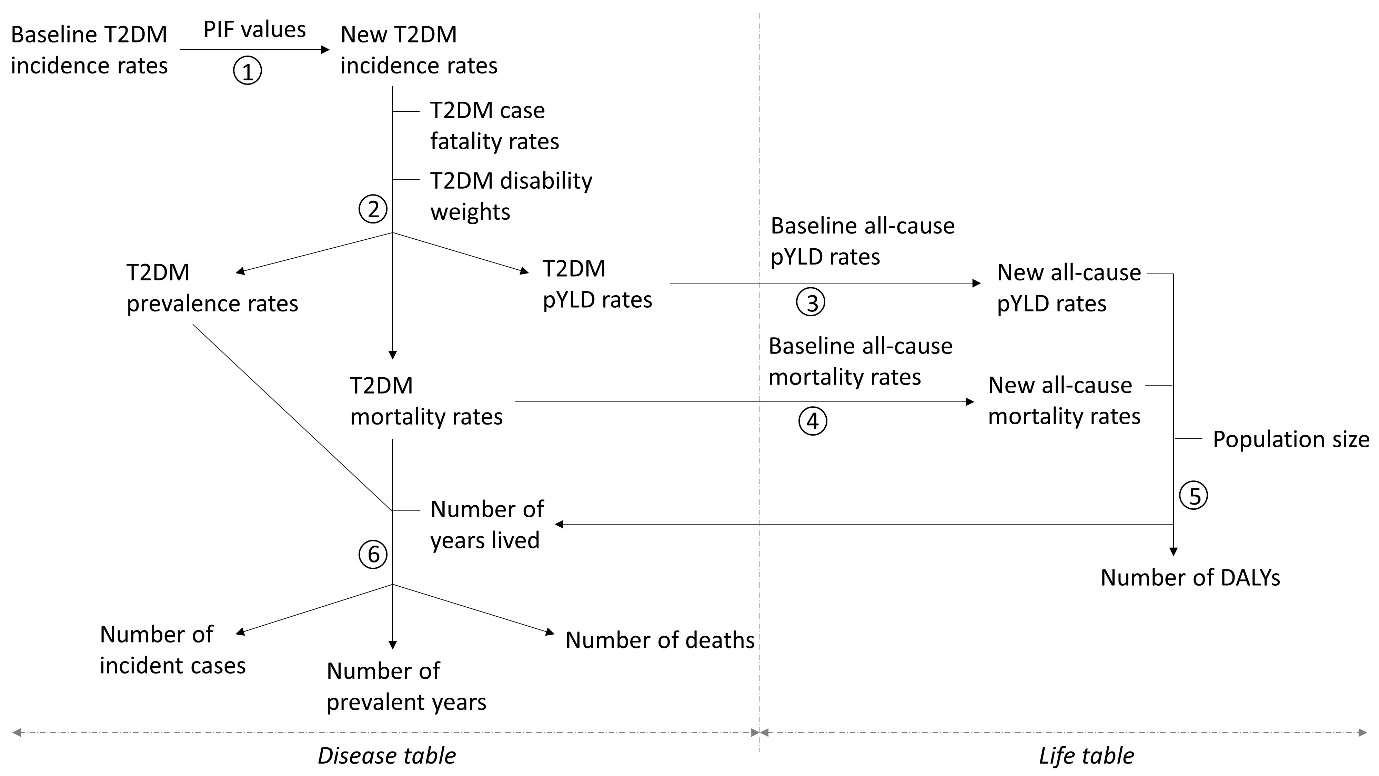


In the disease table the yearly number of individuals in each of the states (i.e., healthy, diseased and dead) are calculated by implementing the following system of differential equations following Barendregt et al. (2003):

|  | $\frac{dS_{a}}{da}=-i_{a}S_{a}$ | (8) |
| --- | --- | --- |
|  | $\frac{dC_{a}}{da}=-f_{a}C_{a}+i_{a}S_{a}$ | (9) |
|  | $\frac{dD_{a}}{da}=f_{a}C_{a}$ | (10) |

In equations (8) to (10), *S_a_* represents the number of healthy individuals, *C_a_* the number of individuals diseased with type 2 diabetes, and *D_a_* the number of individuals dead from type 2 diabetes at age *a*, respectively; *i_a_* and *f_a_* stand for the type 2 diabetes specific incidence and case fatality rates associated with age *a*.

Based on the previous system of equations, the yearly number of individuals found in the three different states can be obtained from equations (15), (16) and (17) following Barendregt et al. (2003) [10]. The latter include the intermediate terms *I_a_*, *q_a_*, *w_a_*, *v_a_*, written as expressions of the transition probabilities *i_a_* and *f_a_* (equations (11) to (14)).

| $I_{a}=i_{a}+f_{a}$ | (11) |
| --- | --- |
| $q_{a}=\sqrt{i_{a}^{2}-2i_{a}f_{a}+f_{a}^{2}}$ | (12) |
| $w_{a}=exp[-0.5\left( I_{a}+q_{a} \right)]$ | (13) |
| $v_{a}=exp[-0.5\left( I_{a}-q_{a} \right)]$ | (14) |
| $S_{a}=\frac{2\left( v_{a}-w_{a} \right)S_{a-1}f_{a}+S_{a-1}[v_{a}\left( q_{a}-I_{a} \right)+w_{a}\left( q_{a}+I_{a} \right)]}{2q_{a}}$ | (15) |
| $C_{a}=-\frac{\left( v_{a}-w_{a} \right)\left\{ 2\left[ f_{a}\left( S_{a-1}+C_{a-1} \right)-I_{a}S_{a-1} \right]-C_{a-1}I_{a} \right\}-C_{a-1}q_{a}(v_{a}+w_{a})}{2q_{a}}$ | (16) |
| $D_{a}=\frac{\left( v_{a}-w_{a} \right)\left\{ 2f_{a}C_{a-1}-I_{a}\left( S_{a-1}+C_{a-1} \right) \right\}-q_{a}\left( S_{a-1}+C_{a-1} \right)\left( v_{a}+w_{a} \right)+2q_{a}(S_{a-1}+C_{a-1}+D_{a-1})}{2q_{a}}$ | (17) |

Once the yearly number of individuals in the healthy and diseased states are computed the person-years at risk *PY_a_*, can be obtained as in equation (18). The latter is subsequently used to derive age specific values for the type 2 diabetes prevalence proportion, *c_a_* (equation (19)) [10]. By knowing the age specific disability weights associated with type 2 diabetes, *DW_a_*, and based on the computed prevalence proportion values, yearly YLD rates, *YLD_a_*, can then be calculated as in equation (20) [18]. Finally, based on the yearly number of person years at risk and the yearly number of dead individuals, age specific type 2 diabetes mortality rates, *b_a_*, can be determined as in equation (21) [10]. Figure 4 summarizes the calculation steps illustrated in equations (11) to (21) for every 5-year cohort.

|  | ${PY}_{a}=0.5(S_{a}+S_{a+1}+C_{a}+C_{a+1})$ | (18) |
| --- | --- | --- |
|  | $c_{a}=0.5({C_{a}+C_{a+1}}/{{PY}_{a}})$ | (19) |
|  | ${YLD}_{a}=c_{a}*{DW}_{a}$ | (20) |
|  | $b_{a}=\frac{D_{a+1}-D_{a}}{{PY}_{a}}$ | (21) |

S4 Figure: Schematic illustrating the calculation steps in the Markov model


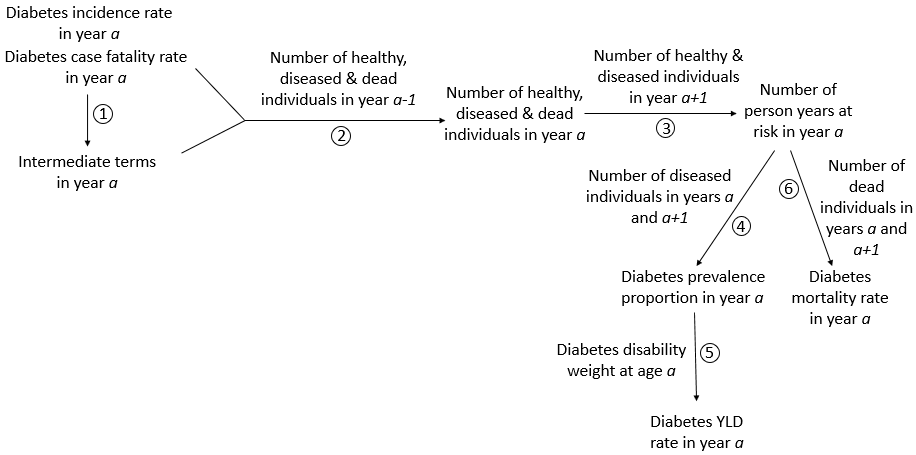


The progression of a cohort is followed via a life table with equal age intervals (*x*, *x+n*). Based on the number of individuals alive at the beginning of an age interval, *I_x_*, and on the probability of death associated with the respective age gap, *_n_q_x_*, the number of deaths occurring during the interval, *_n_d_x_*, can be calculated as in equation (22). The obtained number of deaths, *_n_d_x_*, can then be subtracted from the number of individuals alive, *I_x_*, to yield the number of individuals alive at the beginning of the following age interval, *I_x+n_*, as in equation (23). Notably, when the probability of death associated with an age interval, *_n_q_x_*, is not known, it can be computed based on the mortality rate in the respective age interval, *_n_m_x_*, as in equation (24). Finally, the number of years lived during the age interval, *_n_L_x_*, is computed as the ratio between the number of deaths, *_n_d_x_*, and the mortality rate, *_n_m_x_*, in the respective age gap as in equation (25) [19].

|  | *_n_d_x_* $=$ *_n_q_x_* $*$ *I_x_* | (22) |
| --- | --- | --- |
|  | *I_x+n_*$=$ *I_x_* $-$ *_n_d_x_* | (23) |
|  | *_n_q_x_* $=1-exp(-$ *_n_m_x_*$)$ | (24) |
|  | *_n_L_x_* $=$ *_n_d_x_ / _n_m_x_* | (25) |

The present model uses 1-year life tables in which the age gap *n* is one year. For each population and 5-year cohort, males and females were followed independently through the life table until death or the age of 100 years. A scheme illustrating the calculation steps for every age-sex cohort in each population is shown in Figure 5. Notably, the yearly all-cause mortality rates, *_n_m_x_*, were known for the baseline population only. For the intervention population, these rates were computed in the following way: First, the yearly type 2 diabetes-specific mortality rates obtained for the baseline population in the Markov model were subtracted from the yearly all-cause mortality rates for the same group to obtain the yearly all other causes mortality rates for the baseline population; The latter were then added to the yearly type 2 diabetes-specific mortality rates obtained for the intervention population in the Markov model to derive the yearly all-cause mortality rates for the intervention group.

Once the number of years lived in each age interval was computed for every age-sex cohort for each population, an additional step was to compute the number of DALYs lived in the respective age intervals. The latter was achieved in the model by multiplying the number of years lived in the age interval by the all-cause YLD rate in the considered age gap (Figure 5). As with the all-cause mortality rates, the yearly all-cause YLD rates were known for the baseline population only. For the intervention population, these rates similarly had to be computed in the following way: First, the yearly type 2 diabetes-specific YLD rates obtained for the baseline population in the Markov model were subtracted from the yearly all-cause YLD rates for the same group to obtain the yearly all other causes YLD rates for the baseline population. The latter were then added to the yearly type 2 diabetes-specific YLD rates obtained for the intervention population in the Markov model to derive the yearly all-cause YLD rates for the intervention group.

S5 Figure: Schematic illustrating the calculation steps in the life table


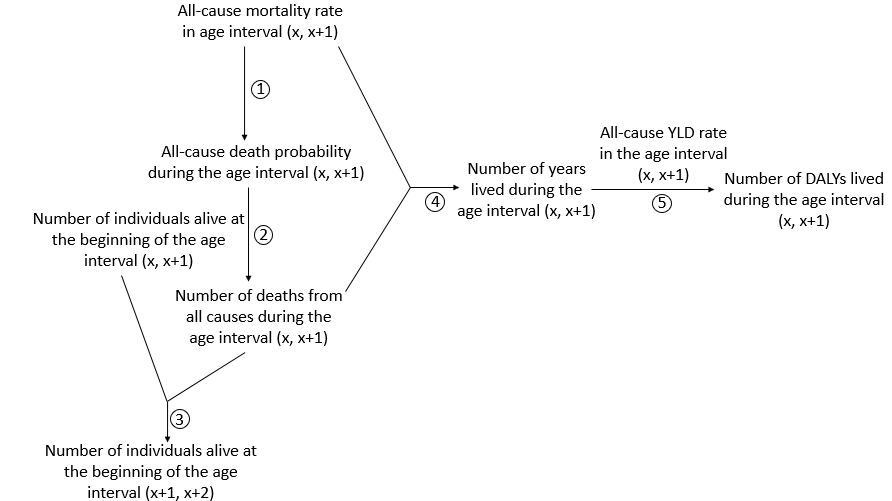


Once the numbers of years lived in all age intervals by each population and age-sex cohort were computed, these could be introduced back in the Markov models to find the main outcome parameters in the analysis. Explicitly, the model derives the yearly numbers of type 2 diabetes-specific incident cases, *N_i_*, prevalent years, *N_p_*, and deaths, *N_d_*, for each age-sex cohort and population as in equations (26) - (28) (Figure 6). In the latter, *i_a_* is the incidence rate, *c_a_* the prevalence proportion, and *b_a_* the mortality rate in year *a*, respectively. Additionally, the number of years lived during the one-year age interval, *_n_L_x_*, corresponds to the age of the cohort in year *a*.

|  | $N_{i}=i_{a}*\left( 1-c_{a} \right)*$ _n_L_x_ | (26) |
| --- | --- | --- |
|  | $N_{p}=c_{a}*$ _n_L_x_ | (27) |
|  | $N_{d}=b_{a}*$ _n_L_x_ | (28) |

Another outcome parameter of interest derived from the Markov model was the yearly cohort-level amount of healthcare costs accrued due to type 2 diabetes cases. This was calculated by multiplying the number of prevalent years, *N_p_*, by the yearly per-capita amount of healthcare costs associated with the disease (Figure 6).

S6 Figure: Schematic illustrating the final steps to obtain the yearly values of the main outcome parameters for every age-sex cohort and population in the model


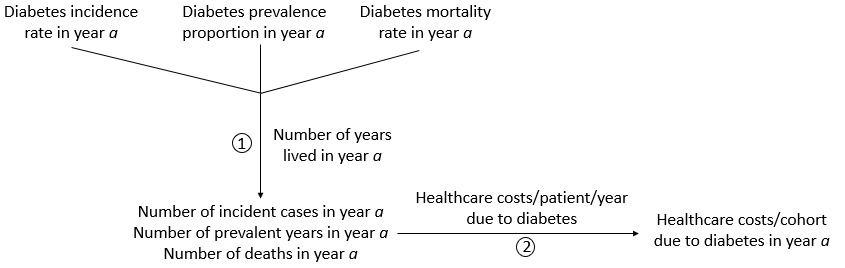


To account for the assumption that the SSBs tax would lead to an increase in life years lived (*L_x_*), and subsequently healthcare costs, an additional step was performed for the cost calculation. Namely, to the previously obtained yearly values for the change in costs after tax, the yearly differences in terms of healthcare costs not related to type 2 diabetes were added. The latter were computed by multiplying the yearly differences in terms of years lived, *L_x_*, by the yearly per-capita healthcare costs not related to type 2 diabetes.

**Appendix 5.** Implementation of uncertainty analyses using Ersatz

Uncertainty analyses in the proportional multi-state life table simulation model were implemented using the free software Ersatz and EpiGearXL, which are available from <http://www.epigear.com/> [17, 20]. This software adds functionality for Monte Carlo analyses and various probability distributions to Microsoft Excel, which is the software the simulation model is implemented in. Parameters that are included in the uncertainty analysis are defined using Ersatz functions, which specify the distribution from which the respective parameter value per Monte Carlo iteration should be drawn. Model outputs are then collected using Ersatz output functions and exported via the Ersatz interface from which mean estimates and uncertainty intervals can be calculated. We used 2,000 iterations to calculate uncertainty in outcomes.

S1 Table: Overview of probability distributions used for model parameters

| **Parameter** | **Distribution** | **Values** |
| --- | --- | --- |
| Change in body weight after tax | Empirical distribution based on Monte Carlo procedure (Appendix 3) | Age- and sex-cohort-specific based on Monte Carlo procedure (Appendix 3) |
| Relative risk to develop type 2 diabetes per BMI-unit | Log-normal | Age- and sex-cohort-specific (Appendix 1) |

**Appendix 6.** Details on modifications of policy modeling assumptions

1. *Adjustment of sugar-sweetened beverage consumption (Modification 3)*

To adjust baseline consumption for sugar-sweetened beverages (SSB) for potential misreporting and out-of-home consumption, we compared yearly consumption in liters per capita calculated with the NVS II data with published aggregate consumption levels from industry reports (Table 1) [1, 21].

To calculate yearly consumption in liters per capita from NVS we computed the survey weighted daily mean consumption in liters (i.e., SSBs = soft drinks & fruit drinks) and multiplied this number by the number of days per year. To calculate the comparison figure from the published aggregate data, we summed up values for the year 2006, which is when NVS II data was collected. We counted the categories soft drinks, diet soft drinks, sports drinks, other soft drinks, coffee & tee drinks and fruit drinks as SSBs (Table 1), which is practically equivalent with our definition of SSBs in NVS II.

To calculate adjustment factors, we divided the beverage-specific per capita consumption per year based on industry data by the respective value based on NVS II data. This procedure resulted in an adjustment factor of 1.86 for SSBs. To adjust beverage consumption, we multiplied individual self-reported SSB with the respective adjustment factor under the simplified assumption that misreporting patterns and measurement biases are the same in all age-sex cohorts. While this is a very rough adjustment procedure it serves the purpose of adjusting average consumptions levels per cohort towards otherwise reported consumption levels per beverage category.

S1 Table: Published consumption values in liters per capita per year from German industry association for non-alcoholic beverages (wafg) [21]

|  | **2004** | **2005** | **2006** | **2007** | **2008** |
| --- | --- | --- | --- | --- | --- |
| **Limonaden (insgesamt) – *soft drinks (total)*** | **80.4** | **81.0** | **81.7** | **84.3** | **83.9** |
| Cola und Cola-Mischgetränke | 33.2 | 35.0 | 33.0 | 33.1 | 30.4 |
| Cola und Cola-Mischgetränke (light) | 7.1 | 6.7 | 7.4 | 7.9 | 8.0 |
| fruchtsafthaltige Limonaden | 24.3 | 24.1 | 28.7 | 33.3 | 35.6 |
| fruchtsafthaltige Limonaden (light) | 1.2 | 1.1 | 1.2 | 1.2 | 1.2 |
| sonstige Limonaden | 13.0 | 12.9 | 10.4 | 7.9 | 7.8 |
| Bitter-Getränke | 1.6 | 1.2 | 1.0 | 0.9 | 0.9 |
| **Diät. Erfrischungsgetränke – *diet soft drinks*** | **2.0** | **1.9** | **2.9** | **3.1** | **3.0** |
| **Mineralstoffgetränke – *sports drinks*** | **1.4** | **1.6** | **1.6** | **1.2** | **1.6** |
| **Brausen & sonst. Erfrischungsgetränke – *other soft drinks*** | **3.7** | **4.6** | **5.4** | **6.5** | **7.2** |
| **Kaffee- und Teegetränke – *coffee & tee drinks*** | **8.4** | **8.0** | **8.2** | **6.6** | **6.9** |
| **Fruchtsaftgetränke (insgesamt) – *fruit drinks*** | **17.5** | **16.2** | **15.7** | **14.8** | **14.3** |
| kohlensäurefreie Fruchtsaftgetränke | 12.0 | 11.3 | 10.7 | 10.0 | 9.8 |
| kohlensäurefreie Fruchtsaftgetränke (light) | 0.8 | 0.6 | 0.7 | 0.7 | 0.6 |
| kohlensäurehaltige Fruchtsaftgetränke | 4.0 | 3.8 | 3.8 | 3.5 | 3.3 |
| kohlensäurehaltige Fruchtsaftgetränke (light) | 0.7 | 0.5 | 0.5 | 0.6 | 0.6 |
| **Fruchtsäfte und -nektare (insgesamt) – *fruit juice & nectars (total)*** | **40.65** | **40.04** | **39.83** | **38.30** | **37.40** |
| Apfelsaft | 12.88 | 12.42 | 12.02 | 11.35 | 9.25 |
| Orangensaft | 9.24 | 8.93 | 8.92 | 8.32 | 8.00 |
| Traubensaft | 1.31 | 1.29 | 1.28 | 1.25 | 1.00 |
| Grapefruitsaft | 0.32 | 0.36 | 0.36 | 0.36 | 0.30 |
| Birnensaft | 0.30 | 0.25 | 0.25 | 0.26 | 0.25 |
| Gemüsesaft/-nektar | 0.99 | 1.35 | 1.36 | 1.35 | 1.20 |
| Zitrusnektar | 7.35 | 7.26 | 7.30 | 7.20 | 8.00 |
| andere Säfte und Fruchtnektare | 8.26 | 8.18 | 8.34 | 8.21 | 9.40 |

1. *Results from the adjustment of price elasticities (Modification 1 & 4b)*

To induce heterogeneity in own- and cross-price elasticities we implemented simplistic adjustments based on other modeling studies and econometrics findings from high-dimensional consumer data. The exact method is described in the main manuscript in its entirety. We here display the results of these adjustments.

*
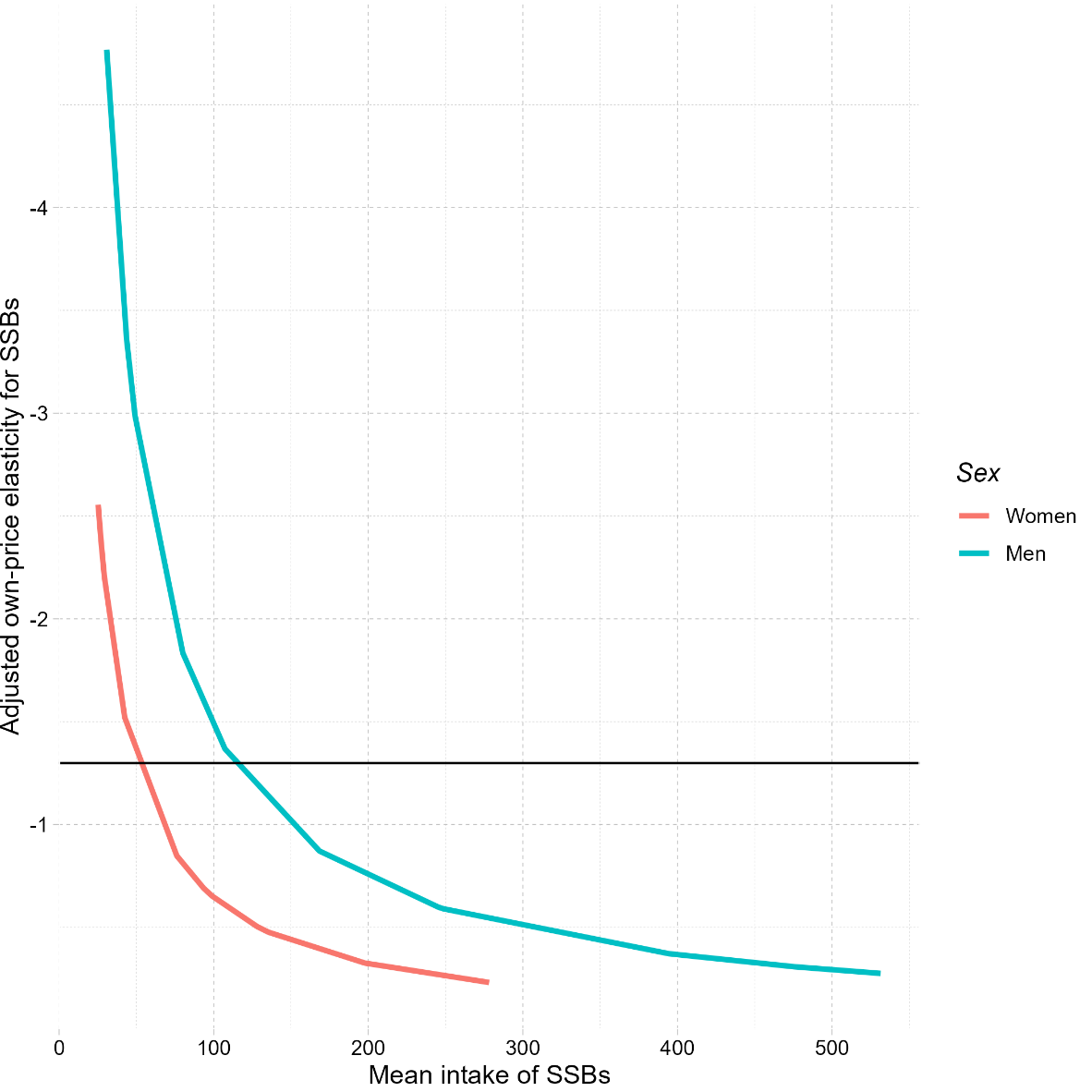
*S1 Figure: Adjusted own-price elasticity for sugar-sweetened beverages

The horizontal black line is the unadjusted own-price elasticity of demand for SSBs from Cabrera-Escobar et al. [2]. Colored lines are the resulting adjusted own-price elasticities for men and women, respectively. Abbreviations: SSB, sugar-sweetened beverages.

*
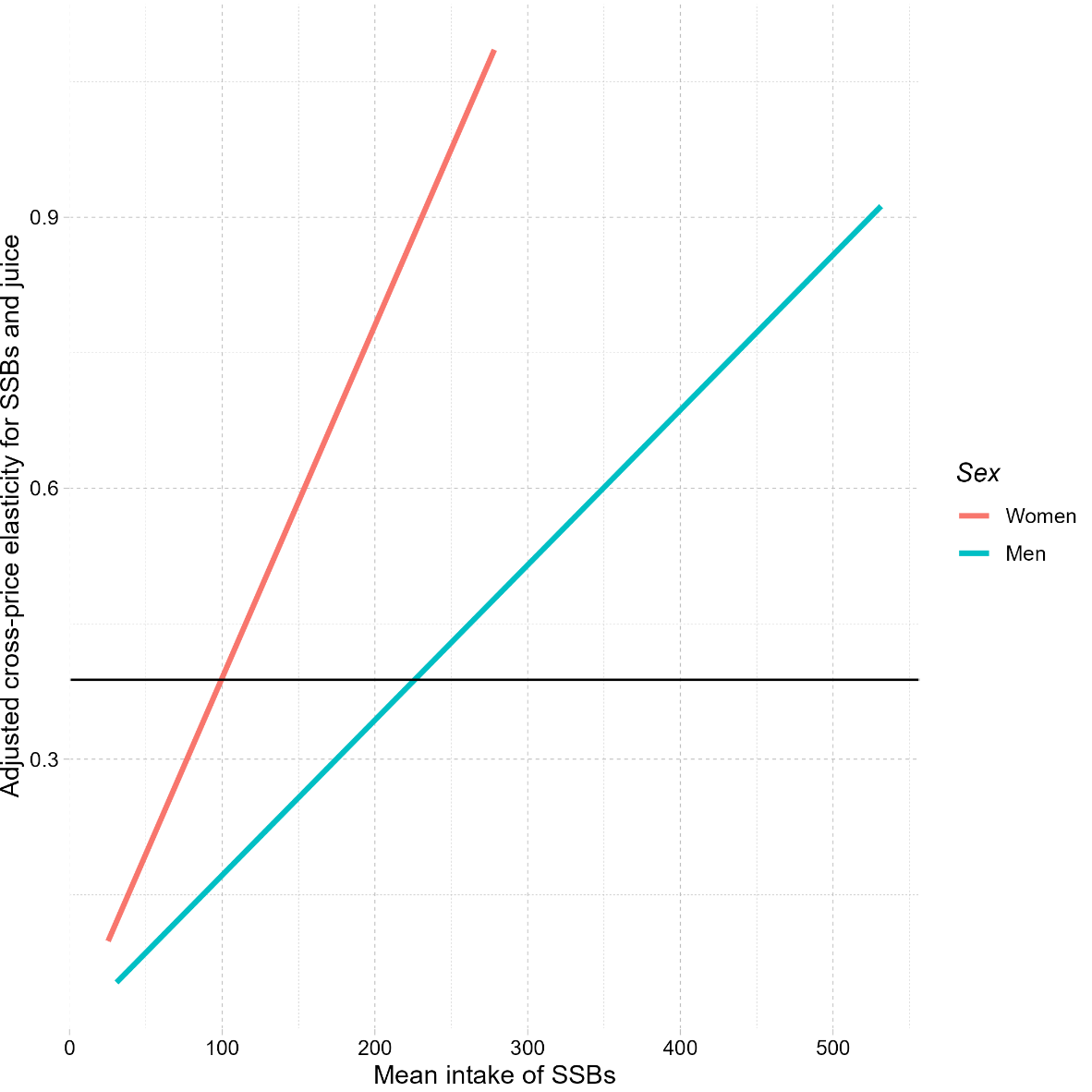
*S2 Figure: Adjusted cross-price elasticity of sugar-sweetened beverages and fruit juice

The horizontal black line is the unadjusted cross-price elasticity of demand for SSBs and fruit juice from Cabrera-Escobar et al. [2]. Colored lines are the resulting adjusted cross-price elasticities for men and women, respectively. Abbreviations: SSB, sugar-sweetened beverages.

**Appendix 7.** Health and economic impact of SSB tax scenarios and assumption modifications

S1 Table: Health and economic impact of SSB taxation on type 2 diabetes in Germany for policy scenarios by sex

| **Main analysis** | **Avoided incident**  **type 2 diabetes cases (95%-UI)** | **Avoided prevalent years lived with type 2 diabetes in thousands (95%-UI)** | **DALYs gained (95%-UI)** | **Healthcare costs saved in € million (95%-UI)** |
| --- | --- | --- | --- | --- |
| Men | 62300 (34200; 99900) | 1630 (943; 2540) | 57000 (33000; 89000) | 1723 (1005; 2678) |
| Women | 24100 (7400; 45900) | 643 (270; 1133) | 20000 (8000; 35000) | 647 (277; 1135) |
|  |  |  |  |  |
| **Scenarios** |  |  |  |  |
| *10% tax rate* |  |  |  |  |
| Men | 22300 (8700; 40100) | 629 (300; 1066) | 22000 (10500; 37300) | 668 (323; 1124) |
| Women | 3200 (5400; 13500) | 151 (33; 382) | 4300 (1500; 11500) | 158 (24; 387) |
| *30% tax rate* |  |  |  |  |
| Men | 101700 (58600; 158600) | 2613 (1569; 3989) | 91400 (54800; 139500) | 2757 (1658; 4201) |
| Women | 44900 (18900; 77000) | 1130 (549; 1884) | 34800 (16600; 58400) | 1132 (554; 1883) |
| *Additional taxation of fruit juice* |  |  |  |  |
| Men | 232000 (161700; 317400) | 5204 (3605; 7131) | 181600 (125700; 248900) | 5425 (3749; 7436) |
| Women | 223700 (153900; 307800) | 4454 (3055; 6098) | 142600 (98100; 195100) | 4351 (2978; 5950) |

S2 Table: Health and economic impact of SSB taxation on type 2 diabetes in Germany for modifications of policy modeling assumptions by sex I

| **Main analysis** | **Avoided incident**  **type 2 diabetes cases (95%-UI)** | **Avoided prevalent years lived with type 2 diabetes in thousands (95%-UI)** | **DALYs gained (95%-UI)** | **Healthcare costs saved in € million (95%-UI)** |
| --- | --- | --- | --- | --- |
| Men | 62300 (34200; 99900) | 1630 (943; 2540) | 57000 (33000; 89000) | 1723 (1005; 2678) |
| Women | 24100 (7400; 45900) | 643 (270; 1133) | 20000 (8000; 35000) | 647 (277; 1135) |
|  |  |  |  |  |
| **Modifications** |  |  |  |  |
| *Modification 1* |  |  |  |  |
| Men | 109200 (58000; 173300) | 1945 (1112; 3025) | 68000 (39000; 106000) | 1985 (1144; 3086) |
| Women | 44600 (18100; 79500) | 830 (359; 1465) | 27000 (12000; 48000) | 805 (351; 1417) |
| *Modification 2* |  |  |  |  |
| Men | 41700 (17800; 73500) | 1048 (456; 1819) | 37000 (16000; 64000) | 1104 (480; 1913) |
| Women | 21700 (8700; 39200) | 513 (204; 917) | 16000 (6000; 29000) | 511 (203; 913) |
| *Modification 3* |  |  |  |  |
| Men | 131500 (78100; 197500) | 3338 (2033; 4956) | 117000 (71000; 173000) | 3517 (2145; 5208) |
| Women | 63100 (30700; 102600) | 1533 (793; 2419) | 47000 (24000; 75000) | 1530 (799; 2413) |

S3 Table: Health and economic impact of SSB taxation on type 2 diabetes in Germany for modifications of policy modeling assumptions by sex II

| **Main analysis** | **Avoided incident**  **type 2 diabetes cases* (95%-UI)** | **Avoided prevalent years lived with type 2 diabetes in thousands* (95%-UI)** | **DALYs gained* (95%-UI)** | **Healthcare costs saved in € million* (95%-UI)** |
| --- | --- | --- | --- | --- |
| Men | 62300 (34200; 99900) | 1630 (943; 2540) | 57000 (33000; 89000) | 1723 (1005; 2678) |
| Women | 24100 (7400; 45900) | 643 (270; 1133) | 20000 (8000; 35000) | 647 (277; 1135) |
|  |  |  |  |  |
| **Modifications** |  |  |  |  |
| *Modification 4a* |  |  |  |  |
| Men | -3200 (-67900; 57200) | 279 (-1078; 1579) | 10000 (-38000; 55000) | 324 (-1083; 1676) |
| Women | -54500 (-132500; 12400) | -828 (-2277; 461) | -28000 (-75000; 14000) | -781 (-2185; 476) |
| *Modification 4b* |  |  |  |  |
| Men | 30200 (-9900; 70800) | 849 (-34; 1813) | 30000 (-1000; 63000) | 901 (-20; 1912) |
| Women | -10500 (-49900; 24600) | -104 (-918; 654) | -4000 (-30000; 20000) | -90 (-890; 658) |
| *Modification 4c* |  |  |  |  |
| Men | 37900 (20200; 63000) | 994 (570; 1606) | 35000 (20000; 56000) | 1051 (604; 1692) |
| Women | 14700 (4400; 29300) | 391 (151; 722) | 12000 (4000; 22000) | 394 (155; 723) |

*Negative values indicate an increase in the disease burden.

**Appendix 8.** Overall health and economic impact for all analyses

S1 Figure: Total reduction in incident cases of type 2 diabetes due to sugar-sweetened beverage taxation in Germany for all analyses


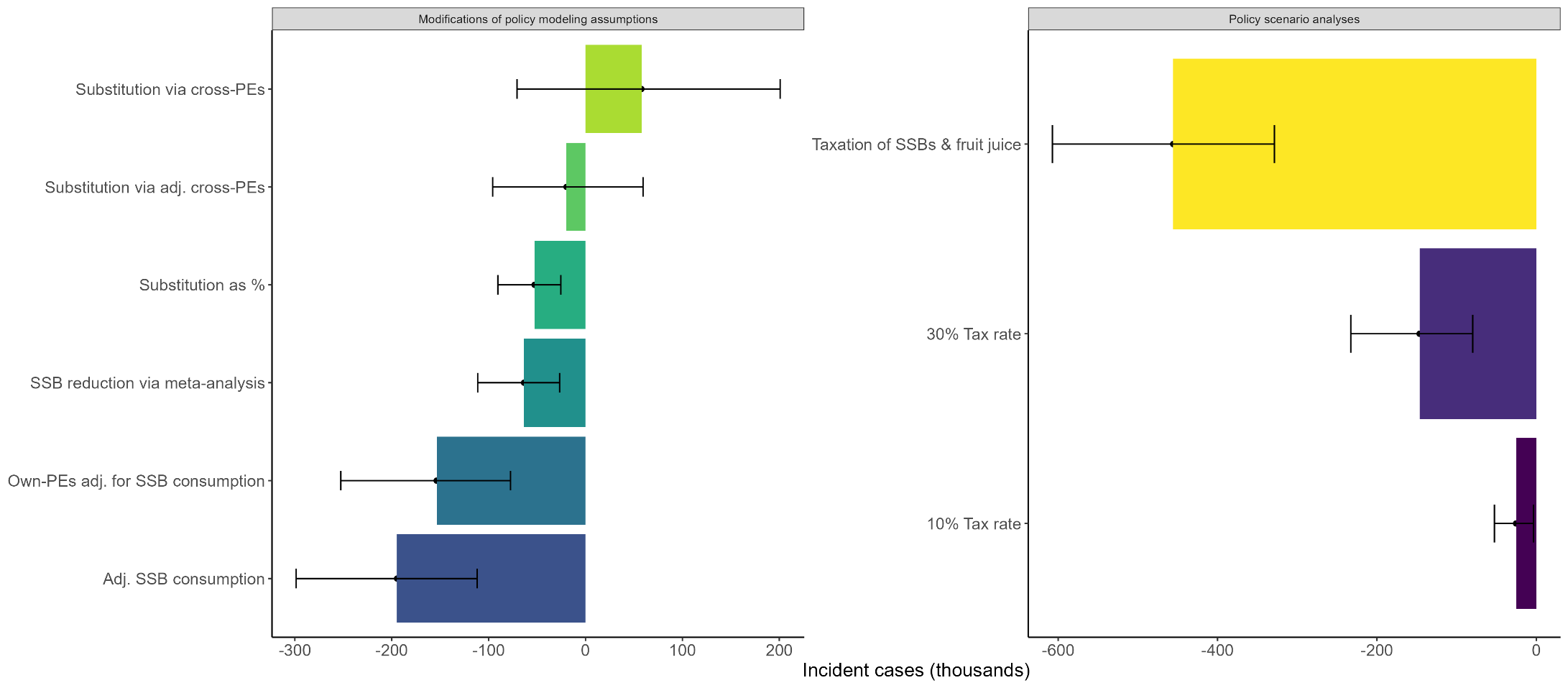


Error bars indicate 95%-uncertainty intervals. Abbreviations: Adj., adjusted; PE, price elasticity; SSB, sugar-sweetened beverages.

S2 Figure: Total reduction of prevalent years lived with type 2 diabetes due to sugar-sweetened beverage taxation in Germany for all analyses


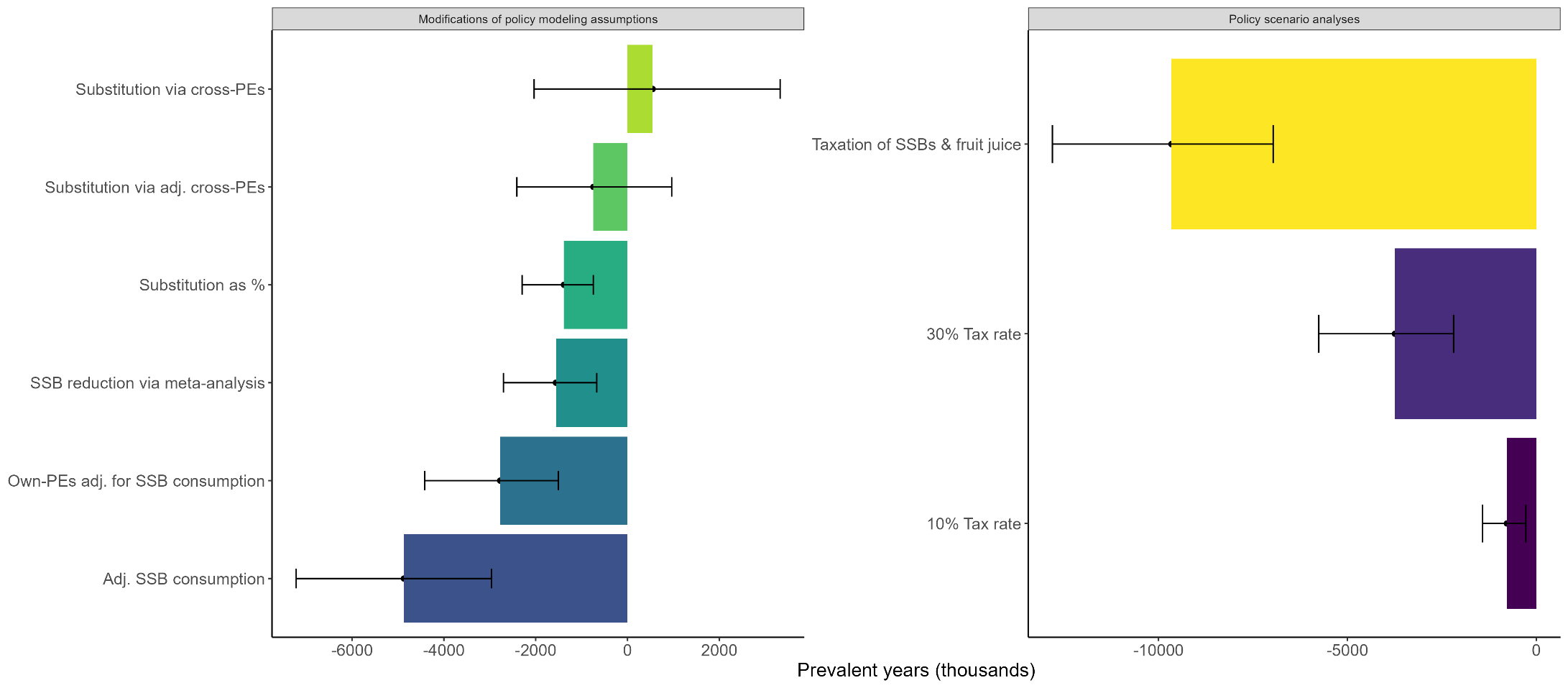


Error bars indicate 95%-uncertainty intervals. Abbreviations: Adj., adjusted; PE, price elasticity; SSB, sugar-sweetened beverages.

S3 Figure: Total gain of disability-adjusted life years from type 2 diabetes due to sugar-sweetened beverage taxation in Germany for all analyses


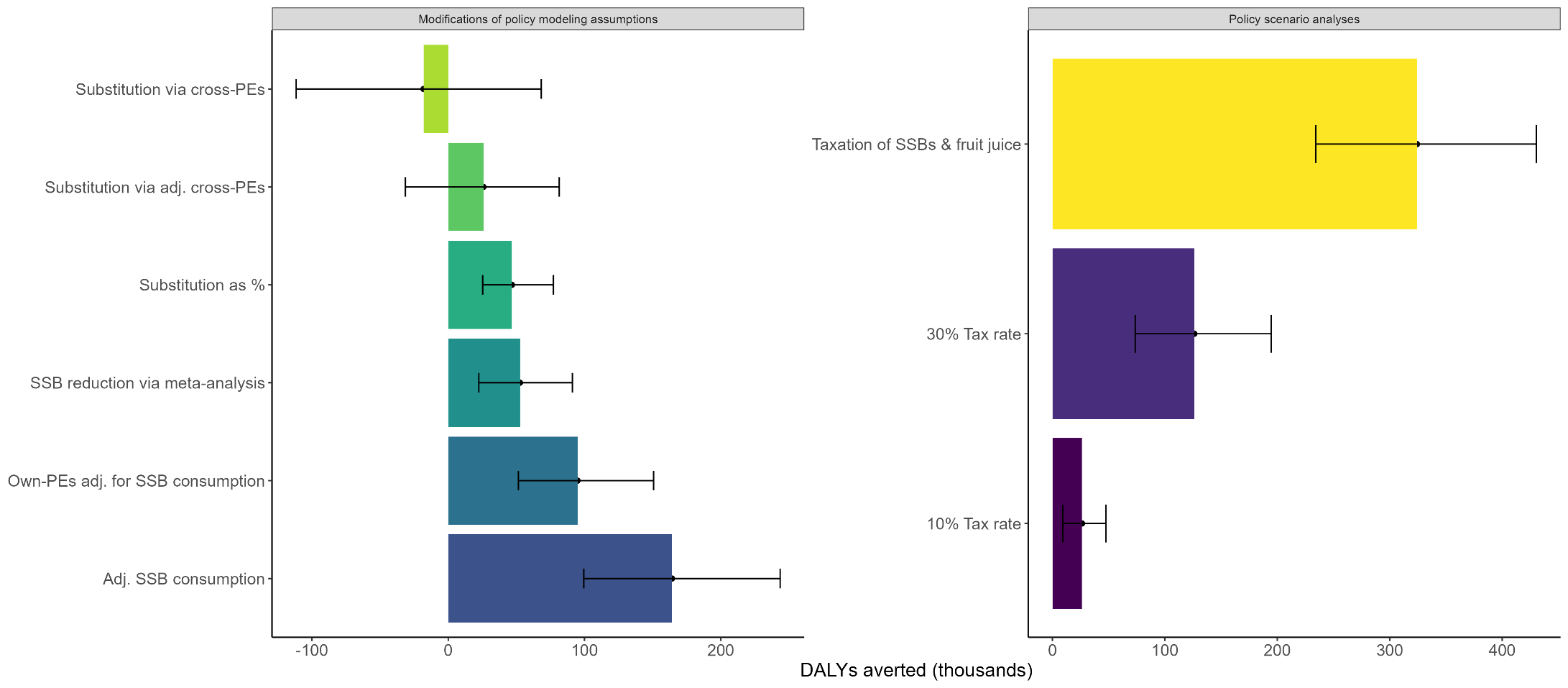


Error bars indicate 95%-uncertainty intervals. Abbreviations: Adj., adjusted; PE, price elasticity; SSB, sugar-sweetened beverages.

S4 Figure: Total reduction of healthcare costs from type 2 diabetes due to sugar-sweetened beverage taxation in Germany for all analyses


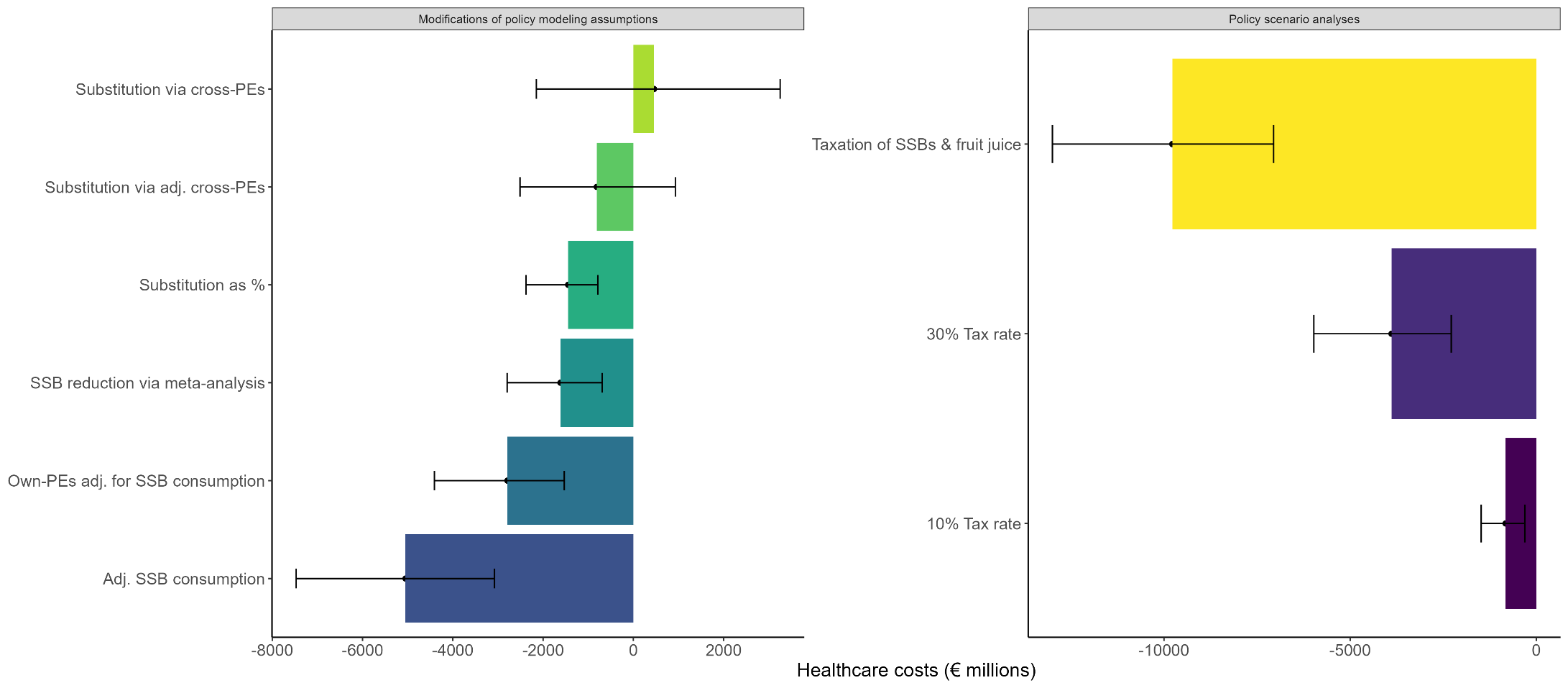


Error bars indicate 95%-uncertainty intervals. Abbreviations: Adj., adjusted; PE, price elasticity; SSB, sugar-sweetened beverages.

**References**

1. Heuer T, Krems C, Moon K, Brombach C, Hoffmann I. Food consumption of adults in Germany: results of the German National Nutrition Survey II based on diet history interviews. Br J Nutr. 2015;113(10):1603-14.

2. Cabrera Escobar MA, Veerman JL, Tollman SM, Bertram MY, Hofman KJ. Evidence that a tax on sugar sweetened beverages reduces the obesity rate: a meta-analysis. BMC public health. 2013;13(1):1072.

3. Predicting the potential health and economic impact of a sugary drink tax in Canada: a modelling study. 2018.

4. Rubner-Institut M. Ernährungsphysiologische Bewertung von Milch und Milchprodukten und ihren Inhaltsstoffen. 2014. DOI:

5. Swinburn BA, Sacks G, Lo SK, Westerterp KR, Rush EC, Rosenbaum M, et al. Estimating the changes in energy flux that characterize the rise in obesity prevalence. Am J Clin Nutr. 2009;89(6):1723-8.

6. Mozaffarian D, Liu J, Sy S, Huang Y, Rehm C, Lee Y, et al. Cost-effectiveness of financial incentives and disincentives for improving food purchases and health through the US Supplemental Nutrition Assistance Program (SNAP): A microsimulation study. PLoS medicine. 2018;15(10):e1002661.

7. GBD Results [Internet]. IHME, University of Washington. 2022. Available from: <https://vizhub.healthdata.org/gbd-results/>.

8. Human Mortality Database [Internet]. Max Planck Institute for Demographic Research (Germany), University of California, Berkeley (USA), and French Institute for Demographic Studies (France). 2022. Available from: [www.mortality.org](file:///G:\Meine%20Ablage\PhD\Publications\2020_SSB_Tax_Germany_MSLT_Andreea\Submission\CRA\Re-Write\www.mortality.org).

9. Schmidt C, Reitzle L, Dress J, Rommel A, Ziese T, Heidemann C. Prevalence and incidence of documented diabetes based on health claims data-reference analysis for diabetes surveillance in Germany. Bundesgesundheitsblatt Gesundheitsforschung Gesundheitsschutz. 2020;63(1):93-102.

10. Barendregt JJ, Van Oortmarssen GJ, Vos T, Murray CJ. A generic model for the assessment of disease epidemiology: the computational basis of DisMod II. Popul Health Metr. 2003;1(1):4.

11. Vos T, Lim SS, Abbafati C, Abbas KM, Abbasi M, Abbasifard M, et al. Global burden of 369 diseases and injuries in 204 countries and territories, 1990–2019: a systematic analysis for the Global Burden of Disease Study 2019. The Lancet. 2020;396(10258):1204-22.

12. Kahm K, Stark R, Laxy M, Schneider U, Leidl R. Assessment of excess medical costs for persons with type 2 diabetes according to age groups: an analysis of German health insurance claims data. Diabetic medicine : a journal of the British Diabetic Association. 2020;37(10):1752-8.

13. Andreyeva T, Marple K, Marinello S, Moore TE, Powell LM. Outcomes Following Taxation of Sugar-Sweetened Beverages: A Systematic Review and Meta-analysis. JAMA Netw Open. 2022;5(6):e2215276.

14. Karter AJ, Nundy S, Parker MM, Moffet HH, Huang ES. Incidence of Remission in Adults With Type 2 Diabetes: The Diabetes &amp; Aging Study. Diabetes care. 2014;37(12):3188-95.

15. Barendregt JJ, Veerman JL. Categorical versus continuous risk factors and the calculation of potential impact fractions. J Epidemiol Community Health. 2010;64(3):209-12.

16. Penman AD, Johnson WD. The changing shape of the body mass index distribution curve in the population: implications for public health policy to reduce the prevalence of adult obesity. Prev Chronic Dis. 2006;3(3):A74.

17. Ersatz. 2016.

18. Collaborators UBoD. The State of US Health, 1990-2010: Burden of Diseases, Injuries, and Risk Factors. JAMA. 2013;310(6):591-606.

19. Barendregt JJ, Van Oortmarssen GJ, Van Hout BA, Van Den Bosch JM, Bonneux L. Coping with multiple morbidity in a life table. Mathematical Population Studies. 1998;7(1):29-49.

20. EpiGearXL. 2016.

21. Getränke WA. Erfrischungsgetränke bleiben beliebt: Wirtschaftsvereinigung Alkoholfreie Getränke; 2010 [Available from: <http://ernaehrungsdenkwerkstatt.de/fileadmin/user_upload/EDWText/TextElemente/Lebensmittel/Wasser-Getraenke/alkoholfreie_Erfrischungsgetraenke_Trend_Afg_Verband_Feb_2010.pdf>.
